# 3-Nitropropionic Acid as a Tool to Study the Mechanisms Involved in Huntington’s Disease: Past, Present and Future

**DOI:** 10.3390/molecules15020878

**Published:** 2010-02-10

**Authors:** Isaac Túnez, Inmaculada Tasset, Verónica Pérez-De La Cruz, Abel Santamaría

**Affiliations:** 1 Departamento de Bioquímica y Biología Molecular, Facultad de Medicina, Instituto Maimónides de Investigaciones Biomédicas de Córdoba (IMIBIC), Universidad de Córdoba, Av. Menéndez Pidal s/n, 14004 Córdoba, Spain; 2 Laboratorio de Aminoácidos Excitadores, Instituto Nacional de Neurología y Neurocirugía “Manuel Velasco Suárez”, México D.F., Mexico

**Keywords:** 3-nitropropionic acid, Huntington’s disease, quinolinic acid, succinate dehydrogenase, transgenic mice models

## Abstract

Huntington’s disease (HD) is an inheritable autosomal-dominant disorder whose causal mechanisms remain unknown. Experimental models have begun to uncover these pathways, thus helping to understand the mechanisms implicated and allowing for the characterization of potential targets for new therapeutic strategies. 3-Nitropropionic acid is known to produce in animals behavioural, biochemical and morphologic changes similar to those occurring in HD. For this reason, this phenotypic model is gaining attention as a valuable tool to mimick this disorder and further developing new therapies. In this review, we will focus on the past and present research of this molecule, to finally bring a perspective on what will be next in this promising field of study.

## 1. Introduction

Research in the field of 3-nitropropionic acid (3-NP) toxicity is extensive, so in pragmatic terms, it is impossible to cover all the studies performed with this toxin up to the present. For this specific reason, we would like to offer our sincere apologies in advance for those relevant studies that were unintentionally omitted in this review. For sure, several groups have made important contributions to this field, so we will try to cover as many relevant studies as possible.

The purposes of this review are: i) to bring the reader into contact with some of the most relevant findings that have served to establish the whole toxic model with a historical perspective, ii) to briefly explore some of the recent research on this field that is offering relevant information to enrich our perspective on this model, and iii) to offer the reader some potential perspectives on those lines of research that will be probably explored through this model to contribute to the understanding of toxic mechanisms involved in neurodegenerative disorders. In particular, the 3-NP toxic model is offering inferential information on those toxic events occurring in Huntington’s disease (HD). For these reason, we will divide this review into three different parts, each one corresponding to a specific chronological phase of research on 3-NP; and start this review with a brief description of this disorder.

## 2. The Past

HD is a neurodegenerative process mainly affecting the basal ganglia in the brain. Symptoms appearing in this disorder have been described for long time (different descriptions can be documented as early as the fourteenth century). Indeed, HD was also known as Saint Vitus’s dance or dancing plague. The disease was first described by Charles Waters as a convulsive disorder, but it was in 1872 [1] when George Huntington formally described it for the first time and referred to as a hereditary chorea. 

HD is catalogued as a rare disease, with a stable prevalence in white populations affecting 5-7 individuals per 100,000 [2,3,4,5,6,7,8,9,10]. The age of onset ranges between 30 and 40, with death occurring after 15–20 years; onset sometimes occurs early in young people at around 20 and evolves over periods of around five years [1,11,12,13,14,15,16,17]. It is also known as an autosomal dominant inheritable neuropathological disorder triggered by excessive repetition of the cytosine-adenine-guanine (CAG) triplet, which encodes glutamine present in protein huntingtin (Htt). This triplet is located in exon 1 at the Huntington gene (HTT), also known as transcript 15 (IT15) [17], located in region 16.3 at the short arm of chromosome 4 (4p16.3). 

The number of triplets expressed by the Htt-encoding gene - and therefore the extension of polyglutamines present in the protein - will determine penetrance, age of onset and probability of transmission to descendants and disease severity.

The symptoms appearing and developing during the course and evolution of HD can be classified into three large groups: i) physical symptoms; ii) cognitive symptoms; and iii) psychiatric symptoms. The first evident symptoms are physical inability accompanied by a wide variety of cognitive-psychiatric alterations [18,19,20,21,22,23,24,25,26,27,28,29,30,31,32].

Although several biochemical, molecular, physiological and anatomical changes in HD have been extensively described, these have yet to be fully established and clarified; nevertheless, numerous findings in recent decades [33,34,35,36] have enabled researchers to put forward different hypotheses about different molecular mechanism potentially occurring in this disorder [32,37,38,39,40,41,42,43].

Different biochemical studies have also revealed the existence of major defects in the energetic metabolism of HD patients characterized by mitochondrial dysfunction. Mitochondria of HD patients are affected by alterations in electron transport chain (ETC) function, in which complexes II and III are affected, prompting a significant decrease in succinate oxidation and ATP synthesis. Complex IV (cytochrome oxidase) is also affected, albeit to a lesser extent. Other defects seem more selectively distributed, such as in the case of complex I (NADH dehydrogenase) and pyruvate dehydrogenase complex [44,45,46,47,48,49,50].

Mitochondrial dysfunction is the main source of reactive oxygen species (ROS). ROS-triggered excitotoxicity also induces massive entry of calcium ions (Ca^2+^) from the extracellular medium, prompting the release of this ion stored in the mitochondrion and endoplasmic reticulum to the cytoplasm, and ultimately resulting in the activation of neuronal nitric oxide synthase or nitric oxide synthase type I (nNOS or NOS-I) with the subsequent release of nitric oxide (NO). In turn, NO is transformed into peroxynitrite (ONOO^─^) after reacting with superoxide anion (O_2_^●─^) from the ECT. These events, together with dopamine (DA) metabolism, create an imbalance between oxidant and antioxidant systems characterized by excessive production of ROS as O_2_^●─^, hydrogen peroxide (H_2_O_2_), ONOO^─^ and a reduction in enzymatic (superoxide dismutase, SOD; glutathione peroxidase, GPx) and non-enzymatic (reduced glutathione, GSH) antioxidant systems, resulting in the appearance of exaggerated oxidative status characterized by macromolecular damage due to oxidative stress (OS). This imbalance promotes typical OS cascades, suchas oxidation of proteins and DNA, and lipid peroxidation. This phenomenon is associated with cellular damage and neuronal death and plays a crucial role in the neurodegenerative process of HD by helping explain the strengthening or intensification of the toxic effect of mHtt [49,50,51,52,53,54,55,56,57,58]. In this regard, one important histopathological finding was the discovery of mHtt protein deposits in the form of inclusion bodies or intraneuronal aggregates in HD [20,35,59]. The mechanism triggering aggregation resulting in selective neuronal dysfunction has not yet been determined. However, it is likely that a conformational change reduces the susceptibility of mHtt to degradation by the ubiquitin-proteasome system, thereby facilitating inclusion formation. It has also been reported that protease-induced mHtt degradation causes the appearance of fragments that facilitate aggregate formation [51,60,61,62,63,64,65,66,67,68]. Thus, through this and other mechanisms, mHtt may affect nuclear and cytoplasmic proteins that regulate transcription factors (e.g. CBP, REST), survival/neurogenesis/apoptosis signalling (e.g. Akt, EGF, p53), mitochondrial function, tumour suppression, vesicle release (e.g., brain-derived neurotrophic factor (BDNF) vesicles), proteolysis (caspases and calpain), protein degradation (ubiquitin-proteasome system), neurotransmissors and axonal transport [28,69,70,71,72,73,74,75,76,77,78,79,80].

Similarly, the generation of models that mimic, to a greater or lesser extent, the HD phenotype and biochemical-molecular and cellular changes in the disease have allowed researchers to better understand the process and perform a clearer and more detailed study of the biomolecular mechanisms participating in and/or facilitating the development of HD. This has also enabled the development of useful models for studying strategies.

### 2.1. The 3-nitropropionic acid (3-NP) model

Nowadays, researchers are able to study the *post-mortem* brains of HD patients and analyse biochemical-molecular parameters in biological media, such as blood and cerebrospinal fluid. Nevertheless, animal models have unquestionable value, despite their limitations, due to the considerable amount of anatomopathological, histopathological, physiopathological, biochemical and molecular data they can provide, thereby allowing researchers to obtain better knowledge of these phenomena and further integration in HD. They are also very useful tools for designing and studying new therapeutic targets, procedures and drugs.

Since the 1970's, different animal models have been developed for studying HD. The first was induced by kainic acid (KA) [81], based on the vulnerability of striatal neurons to excitotoxicity caused by excessive stimulation of excitatory amino acid receptors with further triggering of neuronal death [28,82]. Other models have been developed since then: for instance, the models induced by quinolinic acid (QA), malate (malonic acid; MA) and 3-NP. More recently, transgenic models have also been developed in both rodents (the first transgenic mouse model dates from 1996) [83] and other inferior organisms: the vinegar fly [84] and *Caenorhabditis elegans* [85]. However, the most used models are undoubtedly the rodent and non-human primate models.

The most widely used models for studying neurodegenerative processes in the specific case of HD have been non-genetic models because they are easy to use, control and acquire. Basically, these models induce cell death through excitotoxicity mechanisms (KA and QA) or alteration of mitochondrial metabolism (3-NP and MA). 

The central nervous system is particularly sensitive to variations in energy resources due to the high metabolism of neurons, with alterations in oxidative metabolism clearly representing a risk for the viability of this model. Changes in the availability of energy substrates, such as glucose or oxygen, cause an alteration that affects membrane potentials followed by depolarization [43,86]. In recent decades, many diseases have been associated with energy metabolism impairment, including HD, which exhibits decreases in glucose and oxygen levels in the basal ganglia and cerebral cortex [87].

#### 2.1.1. Treatment with 3-NP and behavioral changes

3-NP is a natural toxin synthesized by fungi (*Aspergillus flavus; Astragalus, Arthrinium*) and plants (*Indigofera endecapylla*); it crosses the blood-brain barrier and therefore, it can be administered systemically. The pioneering study in this field was performed by Chinese researchers and Hamilton and Gould [88,89]. Its administration by means of a subcutaneous osmotic pump or direct subcutaneous injection or intraperitoneal injection are effective methods for systemic infusion of 3-NP, although doses must be adjusted daily according to the weight of the animal since its administration prompts a decrease in the animal’s body weight, up to as much as 20 g [90]. Intrastriatal and intraputaminal infusions are also used.

Different animal species and strains can be used to develop this model with similar profiles of neurotoxicity to those seen in HD brains. The most common of these are rodent models, where the effect is triggered in different murine (CD1, C57BL/6, BALB/c, Sebster/Swiss, 129SvEMS, *etc*.) and rat (Fischer, Lewis, Wistar, *etc*.) strains. Interestingly, the response to this neurotoxin differs according to the species and strain used; doses must therefore be adjusted according to weight, administration period (method and time), species and strain of animal used to obtain the desired biochemical-molecular, cellular and phenotypic changes (acute, subacute and chronic) [44].

Thus, rats are more sensitive to 3-NP treatment than mice. Among different rat species, Fischer rats are the most vulnerable to the toxic action of 3-NP. However, these rats are often not suitable for study due to the difficulty of controlling damage caused by this toxin. In contrast, Lewis rats are ideal for use with 3-NP since they are less sensitive than Fischer rats and respond more stably and consistently to 3-NP in terms of behavioral alterations and lesions [91,92]. Wistar and Sprague-Dawley rats are also sensitive to this agent [44,93,94,95,96,97,98,99,100,101,102,103,104,105,106], developing, despite lower sensitivity to 3-NP, lesions and behavioral alterations of extraordinary value for studying possible routes involved in HD, as well as for testing new therapeutic strategies (Sumarized data in Table 1).

The 3-NP model can mimic and reproduce the hyperkinetic and hypokinetic symptoms of HD, depending on the time and dose administered, thus allowing the initial (or early) and late phases of HD to be evaluated. The administration of 3-NP (10 mg/kg intraperitonelly for more than four doses) induces the onset of similar symptoms to hypokinetic symptoms, while administration in two individual doses diplay similar symptoms to hyperkinetic symptoms (Table 1) [107]. The effects of acute treatment with 3-NP at a maximum dose of 20 mg/kg are observed after the first two injections (), with expression of a phenotype similar to the HD phenotype, although its histopathology is different to that observed in the initial stages of HD, often accompanied by extra-striatal lesions [107,108,109,110].

**Table 1:** Treatment with 3-NP in rats.

Rats	Acute	Sub-chronic and Chronic
**Fischer**	10 mg/kg/day (2–3 days, i.p.)	
	10–30 mg/kg/day (1 day, i.p.)	
**Lewis**	38 mg/kg/day (2 days, s.c.)	50–60mg/kg/day (3–4 days, i.p.)
	15 mg/kg/day (1 day, i.p.)	38 mg/kg/day (5 days, i.p.)
		10–15 mg/kg/day (28 days, i.p.)
**Sprague-Dawley**	30 mg/kg (0.5–4 hours, s.c.)	20 mg/kg/day (3–4 days, i.p.)
		15 mg/kg/day (5 days, i.p.)
**Wistar**	10–20 mg/kg/day (1–4 days, i.p.)	10–20 mg/kg/day (7 days, i.p.)
	20 mg/kg/day (2 days, s.c.)	10 mg/kg/day (twice dose by week/4 weeks, i.p.)
		20, 40, 60 mg/kg/day (9 days, i.p.)

i.p.: intraperitoneally; s.c.: subcutaneous.

Chronic administration of 3-NP at low doses (10 mg/kg/day, 3–6 weeks) [111,112] induces a sustained state of metabolic alterations and some other features similar to those displayed by HD patients. For instance, non-human primates under chronic schedules display lip dystonia and choreiform movements, while prolonged administrations of this toxin (for four months) trigger spontaneous dyskinesias and dystonia [113,114,115,116,117,118]. These results suggest the following limitations in this model: i) the existence of quite different aberrant movements between non-human primates and rats; and ii) the differential organization of basal ganglia once again between primates and rodents, the striatum being formed by two well-defined parts in non-human primates and a single structure in rodents, a phenomenon clearly evidenced by certain events such as conduct, where behavioral changes are different in both animals. Thus, in non-human primates, chronic intoxication with 3-NP triggers a series of movements similar to those observed in HD patients, although these motor alterations have not been reproduced in rats under the same experimental conditions. Moreover, rats treated chronically with 3-NP did not display clear dyskinetic movements resembling chorea [112,113].

Although some motor alterations may constitute resemblances to HD, experimental models must also be able to reconstruct cognitive aspects of this dosorder, such as those of memory and attention alterations. In this regard, it has been shown that at the end of a given 3-NP treatment, non-human primates displayed similar cognitive deficit to that observed in HD individuals [117]. According to Borlongan *et al*. [91], the behavioral changes observed in animals administered with 3-NP may be summarised in three major phases: stage I) sleepiness; stage II) uncoordinated march with sterotypical padding and rolling movements; and stage III) lateral and ventral recumbence [43,113,114].

#### 2.1.2. 3-NP and mitochondria

Despite the fact that 3-NP, a metabolite of 3-nitropropanol, was first described in Chinese children who had eaten contaminated sugar [119], it was first identified a few years earlier after the massive poisoning of cattle in the Western U.S. These animals, after being poisoned with infected legume crops, displayed different motor alterations that evolved towards discoordination and paralysis [120]. 

**Figure 1:**
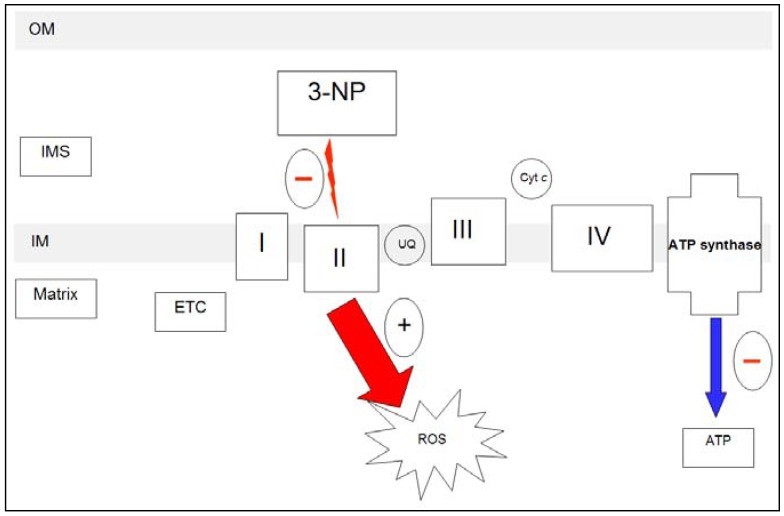
3-NP irreversibly inhibits succinate dehydrogenase (SDH; complex II) of electron transport chain (ETC) and tricarboxylic acid cycle. Schematic representation of the effect of 3-NP on ETC. IM: inner membrane; IMS: Intermembrane space; OM: outer membrane. Complex I: NADH dehydrogenase; Complex III: Cytochrome bc_1 _or cytochrome c reductase; Complex IV: Cytochrome c oxidase; Complex V: ATP synthase.

As mentioned above, 3-NP is a toxin that irreversibly inhibits (suicide inhibitor) the enzyme succinate dehydrogenase (SDH; E.C. 1.3.99.1) [121], which in turn is present in the internal face of the mitochondrial membrane and is responsible for the oxidation of succinate to fumarate. Inhibition of this enzyme invariably leads to neuronal death in caudate and putamen nuclei, triggering severe dystonia in children [120] (Figure 1).

Since the 3-NP-triggered neurodegeneration model mimics the cascade of processes leading to cell death in HD - mainly mitochondrial alteration - as well as some of its histological and pathological characteristics, it has been proposed as a valuable phenotypic model for studying different aspects related with HD and new drugs. 

On the other hand, it is known that alterations in glucose metabolism cause decreased ATP production. Several enzymes involved in the electron transport chain (ETC) and tricaboxylic acid cycle (TCA, Krebs cycle) are indeed, triggered in the course and evolution of HD. Different studies have also shown that aconitase and complex II, III and IV activities are reduced in the striatal nucleus (caudate + putamen) of HD patients [122,123,124]. Therefore, damage to mitochondrial complexes occurs simultaneously to the lost of membrane potential, together with a redistribution of cytochrome c. Some of the studies reporting these alterations have also shown that cyclosporine A, an inhibitor of the permeability transition pore, acts as a neuroprotector against 3-NP. 

3-NP also induces caspase-9 activation, which in turn requires the simultaneous presence of Apaf-1, cytochrome c and ATP. Overall, these data suggest that neuronal death may occur in the presence of intense ATP depletion [125,126,127]. In addition, studies performed in primary neuron cultures show that 3-NP induces the exprssion of different mitochondrial factors associated with apoptosis, including cytochrome c and Smac/DIABLO protein [128].

**Figure 2:**
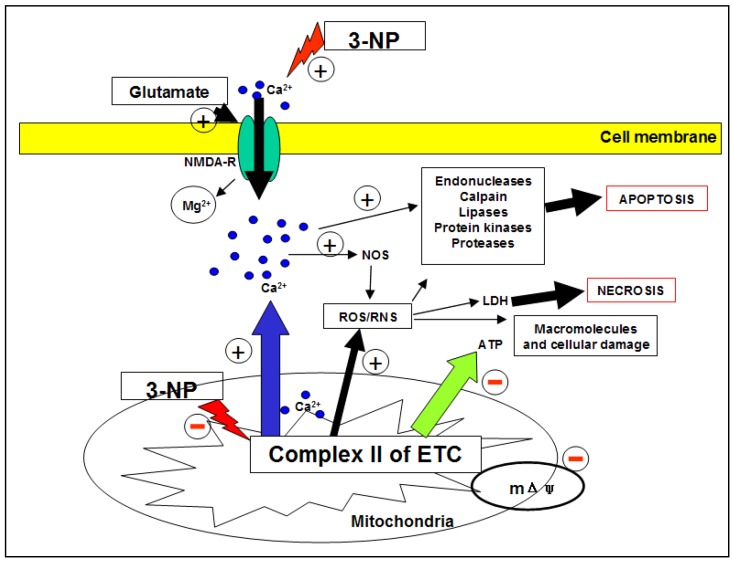
Neurotoxicity by 3-NP: 3-NP promptes complex II inhibition and increased sensibility of NMDA-R (excitotoxicity). 3-NP toxicity affects microglia, astrocytes and neurons, and causes secondary excitotoxicity by making neurons more vulnerable to endogenous basal levels of glutamate, while prompting a reduction of ATP availability. This scenario causes relief of voltage-dependent Mg^2+^ blockade at the NMDA-R pore. In turn, the activation of these receptors leads to massive entry of Ca^2+^ to cytoplasm and further activation of a number of calcium dependent enzymes, including calpains and NOS. Alltogether, these events lead to cell death by different pathways: necrosis and/or apoptosis, depending of intensity of insult and cell type. ETC: Electron transport chain; LDH: Lactate dehydrogenase; mΔψ: Membrane potential; NMDA-R: N-methyl D-aspartate (NMDA) receptor; 3-NP: 3-Nitropropionic acid; RNS: Reactive nitrogen species; ROS: Reactive oxygen species.

In regard to oxidative damage, it is known that the alteration of oxidative metabolism by 3-NP induces oxidative and nitrative stress due to excessive ROS/RNS production and/or depletion of antioxidant systems. Therefore, oxidative damage has been largely linked with neuronal loss in the 3-NP model. Moreover, calcium ions (Ca^2+^) play an important role in this process since its homeostasis is altered by the neurotoxin, which triggers cytosolic increases from internal storages that lead to NOS activation via Ca^2+^/calmodulin, and the subsequent production of NO. Additionally, increased Ca^2+^ concentrations due to the opening of both voltage-gated membrane channels and voltage-gated N-methyl D-aspartate (NMDA) receptor-channel complex trigger excitotoxicity and associated events, including the activation of proteases involved in cell death such as calpains [103,104,129,130,131,132,133,134]. Calpains in turn mediate the degradation of different proteins, including Htt. Interestingly, the activity of this protein is unaffected in areas where 3-NP does not cause cell death [12,128] (Figure 2).

#### 2.1.3. 3-NP and neurotoxicity

Different studies have suggested that glutamatergic innervation due to excitotoxicity plays an important role in 3-NP-induced striatal degeneration. These reports have shown the existence of spontaneous glutamate flow in slices of brain and synaptosomes treated with 3-NP [37,135]. Support to these findings came from Storgaard *et al*. [136], who showed that treatment with inhibitors to recapture glutamate increased the 3-NP-induced neurorotoxicity.

In regard to excitotoxicity, in 1988 Novelli *et al*. [137] established that affecting the energy metabolism in cells may lead to excitotoxicity. These authors demonstrated that oxidative metabolism and Na^+^,K^+^-ATPase dysfunction triggered enhanced neurotoxicity to glutamate [43,111,137,138]. Further studies performed with 3-NP and microdialysis techniques revealed moderate increases in glutamate in rat brains treated with 3-NP. Increased lactate levels have also been reported, indicating that mitochondrial energy disruption is taking place in this model. Altogether, these findings match with those reported by other groups, supporting the idea that 3-NP induces excitotoxicity by turning neurons more sensitive to basal glutamate levels.

Glutamate levels are regulated by sodium-dependent transporters (Na^+^) present in the glia and neurons. The activity of these transporters depends on the transmembrane sodium gradient generated by Na^+^/K^+^ ATPase. Glutamate internalized in the glial cells is metabolised to glutamine and released into the extracellular space, where it is reconverted into glutamate. Therefore, glutamatergic homeostasis mostly depends on the balance and control of all components involved; its alteration may lead to excessive NMDA-R stimulation, prompting their activation and triggering excitotoxicity-induced neuronal death [40,139] (Figure 2). It has also been reported that during ischemia, NO production starts after initial stimulation of NMDA-R, a phenomenon involving neuronal-NOS (nNOS) activation [140].

Of note, 3-NP-induced increases in intracellular Ca^2+^ levels are more intense in astrocytes than in neurons, thus inducing primary astrocyte degradation [94,130,131]. In astroglia, these changes are mediated by the Na^+^-Ca^2+^ exchange system, while other mechanisms are involved in neuronal degeneration [131].

Although most studies on 3-NP cytotoxicity have been focused on neuronal death, other brain cells may also be affected [141]. Regardless astrocytes play a crucial trophic role in neuronal survival, studies on the effect of 3-NP in this regard are still scarce. In 2000, Ohgoh’s research group performed a study in primary neuronal cultures to analyse the effect of astrocytes on neuron vulnerability to 3-NP [142]. This study clearly showed that the presence of astrocytes reduced neuronal vulnerability to the toxicant. Other studies have also reported that, under *in vivo* conditions, modification of the astrocyte phenotype due to over-expression of cytokine ciliary neurotrophic factor (CCNF) protects the striatum from the toxic effects of 3-NP [143]. Deshpande *et al.* [130] showed that chronic intoxication of rats with 3-NP induced striatal damage characterized by lower cell density after hematoxylin-eosin staining of animal brain slices, compared with untreated animals. In addition, loss of immunostained positive cells for glial fibrillary acidic protein (GFAP) in the striatum was revealed by the appearance of empty spaces around the gliosis area. In view of these results, authors reported the existence of astrocyte loss in the lateral area of the damaged striatum. These findings were subsequently confirmed by Villarán *et al.* [144] who found that 3-NP toxicity induced a decrease in GFAP immunoreactivity and microglia activation, determined by immunostaining with OX-6, as well as an increase in the number of apoptotic cells. All these findings revealed and confirmed an important role of astrocytes in neuron survival/death ratio. Moreover, the administration of 3-NP to astrocyte cultures is capable of causing substantial increases in intracellular calcium levels [94,131]; its administration *in vivo *prompts a reduction in the number astrocytes, as well as a loss of white matter and axons, together with loss of oligodendrocytes [141,145,146,147,148]. Although the precise function of astrocytes in the 3-NP-induced toxicity is not completely clear, some reports in scientific literature have shown that these cells are important for removing glutamate from the synaptic cleft and for cell survival. Previous studies have confirmed this observation, showing that 3-NP induces changes in astrocytes prompting a reduction in the release of trophic factors [44]. 

Some enlightening information emerged when 3-NP-induced toxicity in astrocytes was associated with NO. This messenger has been shown to be toxic for CNS in pathological conditions [130]. NO is synthesized by NOS, whose three isoforms are expressed by brain cells:

i) Endothelial NOS (eNOS): also known as type III NOS. This is a calcium-dependent enzyme initially found in the endothelium.
ii) Neuronal NOS (nNOS): also known as type I NOS, present in nerve tissue.
iii) Inducible NOS (iNOS): also known as type II NOS. This enzyme is calcium independent, plays an important role in immune system modulation, and it is regulated by different cytokines. In addition, it produces NO in astrocytes, microglia and macrophages in response to inflammatory reactions.

Microglia, which is activated in the event of intoxication by 3-NP, can also play an important role in the toxic pattern elicited by this molecule. It has been observed that degenerated parts of the striatum are invaded by microglia, the most evident phenomenon from a time and intensity standpoint in the case of acute 3-NP treatment [141]. Microglia activation is accompanied by increased ROS production, thereby allowing these glial cells to participate in the 3-NP-induced neurotoxicity and neurodegeneration [141,149,150].

#### 2.1.4. 3-NP and ROS/RNS

Alteration of mitochondrial activity is associated with abnormally high formation of ROS. ETC enzyme inhibition leads to an increase in electrons released from the mitochondria and the subsequent production of ROS, including O_2_^●─^ and H_2_O_2_. ROS production in turn, alters the balance between oxidants/antioxidants, causing molecular damage that leads to cell death, in a process currently known as OS. Oxidative damage induced by OS affects cell membranes and nucleic acids, as evidenced by the increase in 8-hydroxy-2-deoxiguanosine (8OHdG), carbonylated proteins and lipid peroxides (malondialdehyde, MDA; 4-hydroxynonenals, 4-HDA; thiobarbituric acid reactive substances, TBARS) [151,152].

All these events are triggered by acute or chronic treatment with 3-NP through the inhibition of SDH at TCA and ETC [121,153]. In rats, mice and non-human primates, 3-NP reproduces OS situations similar to those observed in HD [103,104,120,132,154,155,156]. These events are indirectly revealed by the preventive effect shown by the prior or simultaneous administration of different exogenous and endogenous antioxidants [100,101,102,103,104,105,134,157,158,159,160,161,105,134,157]. Together with nitrative, nitrosative or nitrergic stress (NS), OS produces substantial ATP depletion and neuronal death [103,104,153,162] (Figure 2).

3-NP also induces the release of reactive molecules deriving from NO through stimulation of NOS activity. Thus, subsequent induction of NOS leads to NO production. In turn, NO may react with O_2_^●─^ to produce ONOO^─^. The later molecule is characterized by its high cytotoxicity and its capability to induce both protein nitration and hydroxyl radical (^●^OH) formation [163], which is the most toxic and harmful free radical known (Figure 2).

#### 2.1.5. 3-NP, neurochemistry and neuropathology

3-NP induces striatal toxicity, causing degeneration of GABAergic medium spiny neurons in the striatum, resembling those processes observed in HD [154]. When administered systemically under chronic conditions, it causes bilateral, symmetric and selective neuronal degeneration of the lateral striatum [164], being restricted to the dorso-lateral area of the caudate-putamen, and thus mimicking the process that takes place in the dorso-lateral area of the putamen in HD patients [59]. This event suggests that lateral striatal neurons are more susceptible to mitochondrial damage. In contrast, acute administrations of 3-NP produce lesions with more diffuse cellular loss. The neurotoxin induces cell death through necrosis and apoptosis, a couple of processes also observed in the course and evolution of HD [165]. 

3-NP-induced cerebral lesions are more or less specific to the striatum, although other areas located in the hippocampus, thalamus and brain cortex are also affected [91]. These lesions display neuronal loss accompanied by moderate gliosis, decreases in cytochrome oxidase activity and a relative sparing of NADPH diaphorase-positive interneurons and dopaminergic striatal afferents.

In studies with 3-NP, and based on existing data, Bossi *et al*. [166] established and characterized three types of striatal lesions, according to histopathological evidence: i) Type I lesions, described as small lesions distributed randomly in the dorsal striatum with sparing of NADPH-diaphorase neurons; ii) Type II lesions, characterized by greater loss than in type I, with diaphorase neuronal sparing and shrunken islands of cells; and iii) Type III lesions, characterized by neuronal loss in the whole dorsal striatum with slight alteration of the ventral area. Lesions in tyrosine hydroxylase fibers have also been reported [113,114,166,167].

Different studies have shown that DA levels increase after 3-NP administration to animals, which is related with DA release [135]. Indeed, DA represents an additional factor accounting for 3-NP toxicity on GABAergic neurons. Filloux and Townsend reproduced these results and reported that an intrastriatal DA injection triggers a neurotoxic effect, quite similar to the one boosted by simultaneous administration of DA release stimulators such as amphetamines, both in acute and chronic administration models [168]. Additionally, the use of 6-hydroxydopamine (6OHDA), an agent that causes lesions to the sustancia nigra, reduces 3-NP-induced damage [169].

This background prompted Maragos and fellow researchers [170] to propose that endogenous DA contributes to striatal damage caused by excitotoxicity by inhibition of glutamate uptake or by the ROS-independent triggered of mitochondrial complex I. Recently, Villarán *et al.* [144] found that reserpine and alpha-methyl-p-tyrosine-induced DA depletion prevented, at least partially, the 3-NP-induced ROS production in striatal synaptosomes. According to the aforementioned authors, these findings indicate that DA induces mitochondrial ROS production in striatal dopaminergic nerve endings by inhibiting ETC, thus favouring 3-NP toxicity.

Other changes induced by 3-NP in different molecules involved in the neurodegeneration process have been identified. Among them, we can mention: i) consecutive increases in adenosine release upon mitochondrial complex II inhibition [171], ii) decreases in endocannabinoid levels (anadamide and 2-arachydonoyl-glycerol) after 3-NP treatment [172,173], iii) decreases in levels of substance P, enkephalin and choline acetyltransferase [87,91], and iv) increases in levels of somatostatin, neuropeptide Y and neurotensin [87,91] (Table 2).

**Table 2:** Molecular changes induced by 3-NP in rats.

**Adenosine release**	Increased
**ATP production**	Decreased
**Intracellular calcium levels**	Increased
**Caspase-3 activity**	Increased
**Caspase-9 activity**	Increased
**Choline acetyltransferase**	Decreased
**Citochrome c release**	Increased
**Dopamine**	Increased
**Dopamine 3,4-dihydroxyphenylacetic acid (DOPAC)**	Increased
**Endocannabinoids**	Decreased
**Enkephalin**	Decreased
**GABA**	Decreased
**Homovanillic acid (HVA)**	Increased
**LDH**	Increased
**Neuropeptide Y**	Increased
**Neurotensin**	Increased
**NMDA-R**	Increased sensibility to basal levels of glutamate
**NO**	Increased
**ROS production**	Increased
**RNS production**	Increased
**SDH activity**	Decreased
**Somatostatin**	Increased
**Substantia P**	Decreased

#### 2.1.6. 3-NP and death cell

Many mechanisms are involved in striatal MSN degeneration during HD. To determine these mechanisms and processes more accurately, as well as their degree of participation, neuronal toxicity models have been employed, including 3-NP-triggered models [44]. Proposed mechanisms include trophic factors, such as BDNF. 

BDNF is produced by cortical neurons and secreted into the striatum. It is, indeed, essential for striatal neuronal survival and maintenance [174]. Its expression is regulated by sequestering of the transcriptional repressor/neuron-restrictive silencing factor (NSR/REST) in the cytoplasm; in this regard, it is known that mHtt enables the translocation of REST to the nucleus, triggering the suppression of BDNF transcription, accompanied by alterations in neuronal transport [175,176,177,178]. This phenomenon is to be expected, bearing in mind that BDNF is the main support of the striatum and produces a variety of neuromodulatory effects in the brain that are more consistent with local actions than with long-distance retrograde signalling [179]. BDNF and other neurotrophins are also involved in chronic potentiation (LTP) [180]. Noteworthly, decreases in BDNF levels have been reported in the caudate nucleus and putamen of HD patients compared with healthy subjects of the same age and sex [181]. 

BDNF also regulates neuronal plasticity through its trkB receptors [182]. This indicates that neurotrophic factors, especially neurotrophins, nerve growth factor (NGF) and BDNF, all play an important role in regulating the death-survival ratio of adult CNS cells, forming a complex neuroprotection/damage system [183,184]. As a result, some of these neurotrophic factors have been associated with the prevention or delay of apoptosis-induced cell death. 

Hellweg *et al*. [185] showed that damage triggered by 3-NP-induced hypoxia is accompanied by increases in NGF and BDNF in the hippocampus of rats, probably, according to the aforementioned authors, as a neuroprotective response to the repeated action of an inhibited oxidative phosphorilation.

Other interesting factors in the HD animal model are the glial-derived neurotrophic factor (GDNF) and neurturin, given the function of the later in growth, development and trophic support of striatal neurons.

**Figure 3:**
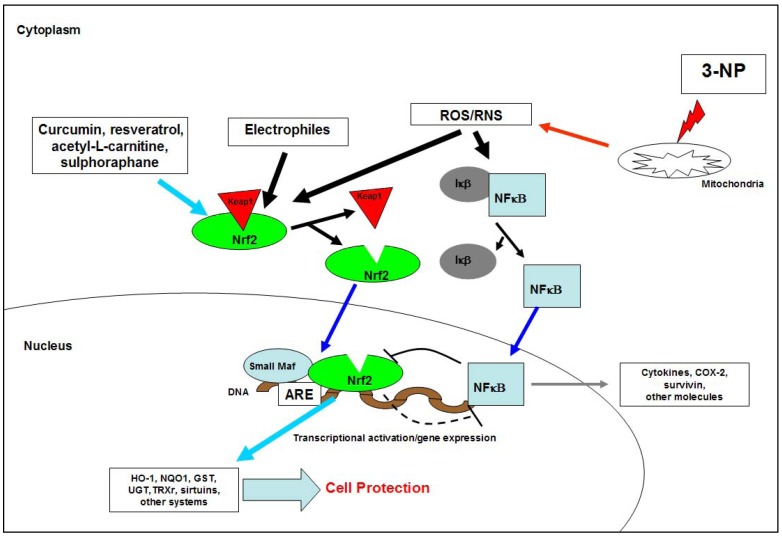
Activation of protective genes by 3-NP. 3-NP triggeres the transcriptional activation of vitagenes expression, which encoded phase II detoxification enzymes, by mean of antioxidant response element (ARE). COX-2: Cyclooxygenase 2; GST: Glutathione S transferase; HO-1: Hemeoxygenase 1; NADPH: Nicotinamide adenine dinucleotide phosphate; 3-NP: 3-Nitropropionic acid; NQ01: NADPH quinine oxidoreductase 1; Nrf2: Nuclear Factor-E2-related factor 2; NFκβ: Nuclear factor kappa beta; ROS. Reactive oxygen species; RNS: Reactive nitrogen species; TRXr: Thioredoxin reductase; UGT: Uridine 5’-diphosphate glucuronosyltransferase. I*kb*: Inhibitor of NFκβ; Keap1: Inhibitor of Nrf2; Small Maf: Transcriptional repressors or transcriptional coactivators.

It is also important to highlight once again the close relationship between neuronal and cell death in general, and ROS/RNS production in cell and molecular death expression, currently known as OS/nitrative stress (OS/NS). As mentioned previously, intoxication with 3-NP triggers intense OS, accompanied by striatal neuronal loss. ROS/RNS activate mechanisms that intend to prevent, protect or recover the tissues from the insult induced by 3-NP. Thus, intoxication with 3-NP with the subsequent ETC complex II inhibition and ROS/RNS production have been linked with activation of the antioxidant response element (ARE), a cis-acting sequence regulating the transcription of several cytoprotective genes. After oxidative insult and subsequent GSH depletion, the nuclear factor erythroid 2-related factor (Nrf2) translocates to the nucleus and dimerises with small Maf proteins (MAF) (family of basic-leucine zipper transcription factors) to form a complex linked to ARE proteins, which are activated to coordinate the expression of genes that counteract the pro-oxidant signals triggered by 3-NP [125,186,187] (Figure 3). ARE inhibits cell death by apoptosis mediated by Fas (signal-transducing adaptor protein that associates with tumour necrosis factor (TNF) receptor complexes), a substrate for proteases similar to caspase-3 and an effector that facilitates cell survival via PK-like ER kinase [188,189,190,191,192].

There is also evidence that 3-NP induces cytochrome c release and activation of both the apoptosis-inducing factor (AIF) and cysteine proteases (caspases) 2, 3 and 8 [192,193,194]. In turn, caspase-2 activation induces the release of mitochondrial cytochrome c and alters the interaction of the later with anionic phospholipids, cardiolipin, thereby increasing the release of this hemoprotein to the external domain. 

Recently, it has been reported that Jun-N-terminal kinase (JNK) activity may play an important role in the pathogenesis of the selective lesion induced by 3-NP [195]. JNK is a kinase belonging to the stress-activated mitogen-activated protein kinase (SAPK), like p38. SAPK is activated by proteins such as apoptosis signal-regulating kinase 1 (Ask1), belonging to the mitogen-activated protein kinase kinase kinase (MAPKKK) family, which in turn are activated by cell stress, in particular by OS [196,197].

Another possible process involved in apoptosis-induced neuronal death has been described by Pelegri *et al*. [198]. This group found that cell cycle activation in brain tissue is one process involving 3-NP-induced neuronal death, reporting neuronal expression of G1 markers (cyclin dependent kinase, CDK: CDK4, CDK2; and cyclin, Cy: CyD1, CyE), and activation of the transcription factor E2F-1 (which interacts directly with retinoblastoma protein and cyclin A, facilitating cell cycle entrance and DNA synthesis) in the striatum after 3-NP treatment. 3-NP has also been shown to reduce the expression of the p27 cellular cycle inhibitor and increase phosphorilation of the retinoblastoma protein [199], as well as activation of CDK5, which regulates calpain activity [200]. Altogether, all these signals point out to one major event: enhanced molecular toxicity and degeneration via OS.

Some studies have shown that 3-NP-induced cytotoxicity is accompanied by elevations of lactate dehydrogenase (LDH) - a characteristic marker of death by necrosis [100,101,102,165,201] -, indicating that this type of cell death is taking place in the toxic insult triggered by molecule, albeit to a lesser extent.

All these data endorse the fact that 3-NP induces neuronal death by both necrosis and apoptosis, probably triggered by excitotoxicity and OS [147,165]. These phenomena may also be associated with an inflammatory response. Seidel’s group showed that 3-NP increased the levels of the intracellular adhesion molecule (ICAM)-1 in a model of murine neuroblastoma cells (Neuro-2a) [202].

The nuclear factor-κB (NFκB), a protein related with the immune system and inflammatory response, has been associated in neurons with the response to excitotoxicity, metabolic stress and OS, and may be responsible for the induction of both pro- and anti-apoptotic genes, depending on the intensity and nature of the stimulus. Recent findings show that this regulatory factor may be involved in the promotion of nNOS transcription; hence, 3-NP would induce nuclear translocation of NFκB and the simultaneously expression of iNOS and nNOS [203] to create a pro-inflammatory scenario. 

#### 2.1.7. 3-NP model: advantages and disadvantages

Although oxidative metabolism inhibitors develop specific HD aspects, since this is a genetic process, their use has a series of disadvantages and advantages [90,204,205,206]:

Disadvantages:

I) mHtt is not produced or folded in metabolic toxic models; cytoplasmic and neuronal inclusions are therefore not observed;
II) the onset of cellular death is progressive and inversely proportional to the number of CAG triplets, a situation (especially the later) not replicated in the metabolic model since cell death is induced immediately by 3-NP through excitotoxicity and metabolic mechanisms not dependent on mHtt;
III) despite the fact that the 3-NP model reproduces different cognitive and behavioral aspects of the HD phenotype, the resemblance of other behavioural aspects, such as suicidal tendencies and obsessive-compulsive conduct, has not been possible. Nonetheless, results obtained recently by our group (unpublished data) show that the administration of a daily dose of 3-NP (10 mg/kg/ip) for three days induces depression and anxiety valued by the forced swimming and open field test, respectively;
IV) the model induced by 3-NP is itself limited to the bioavailability of the toxicant; once metabolized and eliminated, it ceases to have an effect, enabling tissues to respond to the insult.

Advantages:

I) massive cell death induced by the neurotoxin makes it a useful model for studying neurotoxicity phenomena;
II) it is a useful model for analyzing and studying neuroprotective and neurorestoration therapies for HD patients;
III) it is a useful model also for examining the synergic effect of mitochondrial alterations on Htt mutation;
IV) it is,m indeed, useful for studying mechanisms involved in HD pathogenesis such as ROS formation, protease activation, astrogliosis, *etc*.

In summary, only chronic systemic treatment produces motor dysfunctions and striatal lesions that mimic HD histological and neurochemical alterations. This model can also provide important flexibility for studying different stages of HD, as well as neurotoxic processes or other events involving mitochondrial alterations. It may also be useful for testing the efficiency of different treatments at different stages of the disease. However, it seems that genetic models are currently more popular due to their more adequate mimicking of the alterations that take place in HD, although it should not be forgotten that excitotoxic and mitochondrial toxins are often used in transgenic HD models and in *in vitro* studies to evaluate the sensitivity of aberrant genetic material to these toxicants [207]. 

### 2.2. Other Huntington’s disease induced models: Emphasis on QA and facilitating models

Comparisons of the phenotypic model of HD produced by 3-NP with other toxic models is relevant since different points of vie. In first instance, it provides contrasting information on how different toxic mechanisms and events might be participating in the human pathology. This is particularly valid when considering that other phenotypic model, such as those produced by endogenous molecules or neurotoxins, are more clearly related with direct excitotoxicity through NMDA-R over-activation. In this regard, the dissection of the different toxic mechanisms accounting for modeling HD is of major relevance, interesting models combining toxins and mechanisms have been often developed. In addition, it is necessary to mention that some other toxic models might resemble more closely, some alterations seen in HD, so they deserve special attention. This is the particular case of QA.

On the basis of the well described behavioral, morphological, neurochemical and molecular features of HD, different animal models have been designed for experimental purposes. The animal species in which they have been developed mostly comprehend non-human primates and rodents (mice and rats). Most of them are still currently under investigation in regard to how much they resemble HD features, and for sure, they will continue so, since the results obtained this far constitute valuable approaches for the characterization of mechanistic events underlying the degeneration in the human disorder, as well as for the design of novel pharmacological and molecular therapies.

In this point, it is convenient to distinguish between those models produced by administration of toxic agents, either systemically or intracerebrally (phenotypic models), and those produced by the molecular manipulation of genome to obtain an aberrant expression of proteins (transgenic models). The lines of research oriented to use mutant mice as models for HD are based on those molecular alterations leading to enhanced CAG trinucleotide repeat related with mHtt expression [83], the protein likely to be involved in the brain changes observed in HD patients. In this regard, R6/1, R/2, and more recently JAK mutant mice, represent promising tools for the study of the role of multiple CAG repeats and mHtt in neurotoxicity elicited in animals, although the behavioral, neurochemical and morphological information that these models have provided to the comprehension of those toxic mechanisms occurring in HD is still scarce. This is probably why the use of neurotoxins for experimentation still represents a reliable resource for HD modeling since these agents provide important mechanistic information on the noxious events likely to take place in this disorder. Therefore, in this section we will briefly focus in some phenotypic models additional to that produced by 3-NP.

The most relevant phenotypic models historically explored include the intrastriatal infusions of the glutamate analogues KA, ibotenic acid (IA), NMDA, QA, and glutamate itself, to rodents and non-human primates [208,209,210]. Despite some classical studies showed that both KA and IA produced degeneration of striatal neurons, as well as reduction of glutamate decarboxylase (GAD) and choline acetyltransferase (CAT) activities – two hallmarks of HD - in animal models [211,212], these plant-derived agents were simply unable to mimic other specific morphological and neurochemical features of HD. In light of the obvious limitations that these models faced from the begining, the search for new and more accurate models for HD started in the early 80’s. By far, the most prominent of these models, besides of 3-NP, was the one produced by QA.

Also known as 2,3-pyridinedicarboxylic acid, QA is a tryptophan metabolite at the kynurenine pathway [213]. This oxidative metabolic pathway [214] is located in glial cells and produces at least two neuroactive metabolites (QA as excitatory and kynurenic acid as inhibitory) acting on the NMDA-R. Given its endogenous nature, QA itself has been directly implicated as a potential pathogenic factor in HD, since it has been recently demonstrated that neostriatal and cortical levels of this toxicant - along with those of the pro-oxidant metabolite 3-hydroxykynurenic acid – are significantly enhanced in brains from the early low-grade HD [215]. QA has been currently shown to exert selective striatal toxicity by means of excitotoxic, pro-inflammatory and oxidative mechanisms [216,217,218]. In fact, both QA and other metabolites from the kynurenine pathway have been involved in the pathogenesis of neurodegenerative, infectious, inflammatory and non-inflammatory diseases [219,220,221,222,223,224,225,226]. The toxic features leading to the proposal of QA-induced lesions as a model for HD are typically based on its proved capacity to produce a wide variety of events similar to those of the human disorder, including the striatal depletion of the neurotransmitter GABA accompanied by the selective loss of GABAergic neurons, increased levels of cytosolic calcium concentrations, ATP exhaustion, neuronal OS and further massive cell death [227,228,229]. Following its intrastriatal infusion to rodents, QA has also shown to produce moderate hyperkinetic motor alterations that mimic the early symptoms of HD, while 3-NPA produces more intense changes corresponding to both later symptoms and juvenile onset of HD [230]. Although the effects of QA have been largely related with overactivation of NMDA-R, a compelling body of evidence has implicated OS/NS as an integral part of its pattern of toxicity [217,231,232,233,234]. Despite that part of its oxidative component could be a consequence of excitotoxic events, some reports have deal with the notion that this component might also be an independent factor accounting for cell damage. Indubitably, in the next years, the characterization of signaling pathways through transcription factors, as well as proteomic and genomic analysis, will bring enlightening information on the mechanisms associated with QA toxicity and its role in HD.

More recently, an emerging line of research has provided interesting models to study integrative toxic events occurring in neurodegenerative disorders, including HD. These models comprehend the facilitation of excitotoxic events through the impairment of energy metabolism, and are produced by the combination of toxic molecules in different biological systems and under different experimental conditions, thus turning the neuronal cells more vulnerable to “regular” or moderately high concentrations of excitatory agents, further leading to excitotoxic damage by means of indirect overactivation of NMDA-R (“secondary excitotoxicity”), and cell death [235,236,237,238]. Specifically, this kind of toxic events result from the facilitation of NMDA-R since the impairment in energy metabolism decreases the ATP needed to maintain membrane potential, leading cells to sustained depolarization mediated by a lack of ATPases activity, and this process in turn, will produce activation of voltage-gated NMDA-R. A further study supported this concept by testing malonate (a reversible inhibitor of Complex II at the ETC) at sub-umbral concentrations, in the presence of glutamate, NMDA or AMPA) [239]. In that study, neuronal vulnerability to excitotoxicity was found increased. Altogether, these and other evidences strongly point to a tight relationship between energy metabolism and excitotoxicity, two common elements in HD brains [87,108,240,241]. In terms of mechanistic events, it has been often assumed that massive extracellular calcium crossing the NMDA-R-associated channel might be responsible for these alterations. However, Jaquard and coworkers [242] recently demonstrated that the energy impairment induced by 3-NP, accompanied by a moderate action of QA, produced together a synergic increase in striatal degeneration in rats that mainly involved the deregulation of intracellular calcium in absence of NMDA-R hypersensitization, as well as an increase in calpain activity and cell death. For instance, this suggest that in this specific model, in contrast to other models already mentioned, the mechanisms underlying toxicity might imply different events and signaling pathways, further evoking toxic patterns not only different to other combined models, but also different to the individual models produced by these toxicants (QA and 3-NP). This consideration is under current investigation, and some supporting evidence has been recently collected from a published study demonstrating that intracellular calcium—More than extracellular—is responsible for the oxidative damage to membrane lipids produced by this combined paradigm in synaptic membranes [162]. In that study, an active role of intracellular calcium was evidenced through the use of a calcium chelating agent, BAPTA-AM. Its efficacy was compared with conditions of either available or deprived extracellular calcium in the incubation media.

QA has also been tested in the presence of other energy depletion inducers, such as malonate, producing an increase in the volume of lesion in animals infused with these toxicants [243]. Although the results of this interesting report suggested that the toxic synergism in this model was mediated by NMDA-R sensitization, the obvious differences in regard to the experimental conditions employed in this study and others should be considered. For instance, in contrast to Jacquard’s group, the use of malonate in QA-lesioned animals can produce distinct effects as the first is a reversible inhibitor of succinate dehydrogenase. In addition, several NMDA-R antagonists need to be tested to assess the dependency of the toxic events on NMDA-R. In this regard, testing only MK-801 represents a limitation for the study since this antagonist often produces some non-specific effects due to extensive pharmacological interactions. Altogether, these and other issues might be accounting for the differential results obtained as compared with those of Jaquard’s group and ours.

Derived from this evidence, a crucial question persists: why the use of 3-NP plus QA seems to produce different effects in comparison with other models of facilitated excitotoxicity where other glutamate analogues or other energy depletion inducers were employed? A precise explanation for these differences is in course of being developed, but in the meantime we hypothesize that the combination of some factors evoked by these toxicants may be accounting for the specific effects obtained in injury animals and brain preparations. Among these factors we could speculate on the limited potency of QA as an excitotoxic molecule - which is suggestive of other toxic mechanisms recruited -, its stimulated synthesis by glial cells during neurodegeneration (the potential contribution of glial cells to the toxic elements of these agents, either as substrates or as inducers), the considerable inflammatory response triggered by this agent [244], the potential involvement of other toxic kynurenine pathway metabolites in its pattern of toxicity [245], the irreversible inhibitory action of 3-NP, *etc*. In summary, the many toxic mechanisms exhibited by QA, together with the specificity of 3-NP, can be contributing to generate this peculiar model.

Finally, an interesting alternative to explain how these two toxicants together can produce selective mechanisms of damage has emerged since it has been recently proposed that the 3-NPA-induced secondary excitotoxicity is mediated by a component at the NMDA-R that is resistant to antagonists acting at the glycine co-agonist site [246]. If this effect is recruiting selective actions of QA in an independent manner of NMDA-R, or even avoiding the effects of QA in a combined model, is a question that remains to be elucidated in further studies. Meanwhile, the information that this combined model can provide for the characterization of toxic events taking place in HD phenotypic models will be of major relevance in the next years.

## 3. The Present

This far, we have described evidence from the past (old and recent) that served to build what now can be considered the general concept of 3-NP as a toxic tool to resemble some important features of HD. In this section, we will briefly describe the new approaches that several research groups are currently exploring using this model to direct research toward new horizons. Once again, if some relevant report is unintentionally omitted, this was entirely due to the space limitations of this review. 

OS remains as a major expression in this toxic model, as well as a key target to ameliorate nerve tissue damage and the subsequent source of therapeutic designs. Testing the effects of different natural and synthetic molecules with antioxidant properties against 3-NP-induced toxicity is, today more than ever, a valuable approach to characterize this model. In this regard, it has been recently reported that the natural xanthone α-mangostin (isolated from mangosteen fruit) possesses antiperoxidative properties against 3-NP when tested in both rat brain homogenates and synaptosomal P2 fractions [247]. Another report dealing with an antioxidant strategy demonstrated the antiperoxidative and protective effects that an extract of *Valeriana officinalis* exerted on this model in rat brain homogenates [248]. These approaches are relevant since demonstrate that targeting oxidative damage to lipids may result in structural and functional preservation of nerve tissue. Cyclosporine, an immunodepressant, was recently shown to exert neuroprotective and antioxidant effects in an *in vivo* model of 3-NP toxicity in the striatum, cortex and hippocampus of rats [249]. The proposed mechanism by which cyclosporine produced these effects revealed that 3-NP affected the glutathione redox balance in these regions through stimulating NO formation. Another mechanism attributed to 3-NP as part of its toxic pattern is its proved capacity to exert oxidative damage to proteins. In this regard, 3-NP was shown to induce oxidative modification of alpha-synuclein in a transgenic mice model expressing human alpha-synuclein [250]. Enhanced levels of oxidized and nitrated alpha-synuclein were correlated with neurological deficits in these mice. In addition, the active contribution of striatal dopamine to the OS produced in the HD model by 3-NP in rats has been established [251]. According to this report, dopamine is inducing hydroxyl radical formation, which in turn, contributes to the OS and neurotoxicity evoked by 3-NP, potentiating its effect. Moreover, OS, neurochemical (dopamine metabolism and Heat-shock protein 72 expression) and neurotoxic markers of 3-NP-induced brain damge seem to be all sensitive to thermal modulation, since hyperthermia induced in rats turn the animals more resitant to the toxic insult of this molecule [252], by mechanisms still to be explored. Other drugs potentially acting as antioxidants and neuroprotectants, and recently reported in this model are the cholinesterase inhibitor rivastigmine [48], the combination of the pro-bioenergetics coenzyme Q_10_ plus creatine [253], the sesame seeds extract sesamol [254], lycopene and epigallocatechin-3-gallate [255], the *Withania somnifera* root extract [256], the flavonoid kaempferol - acting partially as antioxidant, preventing calpain activation and creatine kinase preservation inactivation - [257], hesperidin and naringin – acting by potentiation of NOS inhibition - [57], the potent free radical chain breaking antioxidant Trolox^®^ [258], and *tert*-butylhydroquinone and the corresponding induction of antioxidant phase 2 enzymes through the Nrf2-ARE pathway [259].

Indubitably, as judging by this cumulative body of evidence, several antioxidants may exert preventive actions if the CNS under 3-NP attack. Recently, we have develope a particular interest in characterizing the protective properties of antioxidant and energy precursor agents after the toxic insult with 3-NP or QA has begun. In other words, we have explored whether agents such as L-carnitine, when given as post-treatment to rats receiving this toxicants, are able to rescue animals from OS, mitocondrial dysfunction and other toxic features [260]. This far, our experiments have revealed that rescue of striatal tissue and nerve terminals is still possible only after a brief period of time (up to 3 h post-insult), suggesting that some toxic elements evoked by these agents are reversible within a limited time frame. This opens new research lines oriented to characterize those key events that can be counteracted in a short time after initiated tissue damage, as well as the design of therapies acting whitin this time frame. In a near future, these studies will focus specifically on the evaluation of resuce in different functional markers of neural transmission.

Other investigations have enhanced the broad spectrum of 3-NP actions in the Nervous System. For instance, 3-NP has been recently employed as a tool to produce a mouse model of spiral ligament degeneration for studing the pathogenesis of sensorineural hearing loss [261]. Cochleae injected with 3-NP successfully reproduced this pathology in several terms. 

3-NP has been used also for exploring the modulation of apoptotic pathways. Histone deacetylase inhibitors (HDACIs) were shown to prevent p53-dependent and p53-independent BAX-mediated neuronal apoptosis by different mechanisms. In particular, when postnatal cortical neurons are challenged with 3-NP and other toxins, they revealed specific pathways since HDACIs prevented caspase-3 cleavage in a mechanism involving Bax, but not p53 [262]. Moreover, the toxic inhibition of Complex II at ETC induced by 3NP has been recently shown to be responsible for mitochondrial fragmentation and neuronal cell death via NMDA- and ROS-dependent pathway [263]. Derived for this interesting study, authors concluded that mitochondrial fission is the result of secondary excitotoxicity and OS/NS, but not derived from energy deficit. Furthermore, although discarded in some studies, p53 has been involved in other reports as an important mediator of cell damage, specifically as a factor inducing mitochondria dysfunction-triggered autophagy activation and apoptotic cell death in rat striatum lesioned with 3-NP [264].

A novel mechanism of 3-NP-induced cell stress and death was recently reported, in which structural and functional changes of astrocyte cytochrome c oxidase (COX) may be accounting for neuronal cell death [265]. According to authors, 3-NP-induced upregulation of COX isoform IV-2 cause increased enzyme activity and cell death at the expense of elevated mitochondrial peroxide production. 

Neurochemical alterations by 3-NP are currently investigated. A recent report shows that *in vivo* dopamine release – measured by fast-scan cyclic voltametry -, is decreased in this toxic model in rats, in contrast with what is observed in transgenic models [266]. Still, the implications of these findings deserve more exploration since it has been mentioned in this review that some reports highlight the contribution of dopamine in 3-NP toxicity. GABAergic system also suffers, in a differential manner, the insults of different toxicants. GABAergic striatal neurons were recently shown to exhibit caspase-independent, mitochondrially mediated programmed cell death, in a study evidencing that 3-NP elicited a mixed profile of caspase and calpain activation [267]. 

Protective strategies based on neurotrophic factors are also under current investigation. BDNF was shown to exert protective effects in the 3-NP toxic model in cortical neurons [268] thorugh different potential mechanisms. The same group almost simultaneously reported that sonic hedgehog (a morphogen critical for embiogenesis)-mediated BDNF-induced neuroprotection in the rodent 3-NP toxic model is involved in this paradigm [269]. Another explanation for the protective actions of BDNF in this model was offered quite recently, since evidence was collected suggesting that BDNF decreases the levels of the pro-apoptotic protein BIM in mitochondrial and cortical cell lysates through the activation of MEK1/2 pathway [270]. A further novel strategy against 3-NP recently described consists of the use of the brain uncoupling protein UCP4 to attenuate 3-NP-induced cell death by bioenergetic adaptation, further leading to cell survival [271]. The proposed mechanism of protection involves the pharmacological inhibition of extracellular signal-regulated kinases (ERKs) by UCP4, thus limiting the glucose utilization dependency and reducing the activation of cAMP-responsive element binding (CREB) protein. It is therefore inferred that ERKs are crucial for 3-NP toxicity. 

This is as far as this review can go, but for sure several other studies are about to appear in literature, bringing important clues on the mechanisms exerted by this toxin and the modulation of its toxic effects. Nonetheless, we believe that we have cover different interesting recent approaches to this field of research. 

## 4. The Future (Conclusion)

On the basis of the evidence recently collected and already described in this review, the prediction of the lines that this fascinating field of research will follow in the next years is relatively easy. At least five major lines are predicted: i) in first place, it is expected that this molecule will be intensively employed as a tool to explore and resemble the metabolic disturbances linked with depleted energy or altered glucose management in the brain; ii) intense research on pro-oxidant mechanisms evoked by 3-NP at biochemical and molecular levels, its interaction with biomolecules to produce aberrant oxidized or nitrated proteins, as well as the potential protective actions of different antioxidants, will be also part of future research; iii) more specific molecular approaches, either directed to use this agent in transgenic models, exploring the transcriptional and transductional signaling associated with its induced toxicity, or the characterization of its pattern of gene and protein expressions through genomic and proteomic studies, will be investigated; iv) behavioral alterations evoked by 3-NP since different perspectives will be explored; and finally, v) its use in combined paradigms to produce more complete and accurate models of HD or other neurodegenerative disorders will be estimated. Altogether, these lines will bring more enlightening information on those mechanisms involved in the pattern of action of this toxicant in the CNS. In the meantime, we can conclude this review emphasizing those properties that had served for modeling one of the most intriguing disorders in then CNS, Huntington’s disease. As long as one molecule may be able to mimic behavioral, biochemical, morphologic and molecular aspects of this pathology in animal models, it will remain in use as a valuable tool for biomedical research for long time. 

## References

[1] Gonzalez-Alegre P., Afifi A.K. (2006). Clinical characteristics of childhood-onset (juvenile) Huntington disease: Report of 12 patients and review of the literature. J. Child. Neurol..

[2] Avila-Giron R. (1973). Medical and social aspects of Huntington`s chorea in the state of Zulia, Venezuela. Adv. Neurol..

[3] González-Ferrer S., Pineda-Bernal L., Delgado-Luengo W., Villalobos-Cabrera H. (2004). Medical genetics in Zulia, a State of Venezuela. Community Genet..

[4] Okun M.S., Thommi N. (2004). Americo Negrette (1924 to 2003): Diagnosing Huntington disease in Venezuela. Neurology.

[5] Paradisi I., Hernández A., Arias S. (2008). Huntington disease mutation in Venezuela: Age of onset, haplotype analyses and geographic aggregation. J. Hum. Genet..

[6] Pridmore S., Cook A., McCormick G., West A. (1995). Trinucleotide expansion in Tasmanian HD families. Aust. N Z J. Psychiat..

[7] Pridmore S.A. The large Huntington’s disease family of Tasmania. Med. J. Aust..

[8] Pridmore S.A. The prevalence of Huntington’s disease in Tasmania. Med. J. Aust..

[9] Wright H.H., Still C.N., Abramson R.K. (1981). Huntington’s disease in black kindreds in South Carolina. Arch. Neurol..

[10] Young A.B., Shoulson I., Penney J.B., Starosta-Rubinstein S., Gomez F., Travers H., Ramos-Arroyo M.A., Snodgrass S.R., Bonilla E., Moreno H., Wexler N.S. (1986). Huntington`s disease in Venezuela: Neurologic features and functional decline. Neurology.

[11] Aubeeluck A., Brewer H. (2008). Huntington’s disease. Part 2: Treatment and management issues in juvenile HD. Br. J. Nurs..

[12] Biglan K., Shoulson I. (2007). Juvenile-onset Huntington disease: A matter of perspective. Arch. Neurol..

[13] Foroud T., Gray J., Ivashina J., Conneally P.M. (1999). Differences in duration of Huntington’s disease based on age at onset. J. Neurol. Neurosurg. Psychiat..

[14] Leegwater J., Jang-Ho J. (2004). The paradigm of Huntington’s disease: Therapeutic opportunities in neurodegeneration. Am. Soc. Exp. Neuro.Ther..

[15] Nance M.A., Myers R.H. (2001). Juvenile onset Huntington’s disease—clinical and research perspectives. Ment. Retard Dev. Disabil. Res. Rev..

[16] Ribaï P., Nguyen K., Hahn-Barma V., Gourfinkel-An I., Vidailhet M., Legout A., Dodé C., Brice A., Dürr A. (2007). Psychiatric and cognitive difficulties as indicators of juvenile huntington disease onset in 29 patients. Arch. Neurol..

[17] The Huntington’s Disease Collaborative Research Group (1993). A novel gene containing a trinucleotide repeat that is expanded and unstable on Huntington`s disease chromosomes. Cell.

[18] Arnulf I., Nielsen J., Lohmann E., Schiefer J., Wild E., Jennum P., Konofal E., Walker M., Oudiette D., Tabrizi S., Durr A. (2008). Rapid eye movement sleep disturbances in Huntington disease. Arch. Neurol..

[19] Björkqvist M., Wild E.J., Thiele J., Silvestroni A., Andre R., Lahiri N., Raibon E., Lee R.V., Benn C.L., Soulet D., Magnusson A., Woodman B., Landles C., Pouladi M.A., Hayden M.R., Khalili-Shirazi A., Lowdell M.W., Brundin P., Bate G.P., Leavitt B.R., Möller T., Tabrizi S.J. (2008). A novel pathogenic pathway of immune activation detectable before clinical onset in Huntington’s disease. J. Exp. Med..

[20] Eskenazi B.R., Wilson-Rich N.S., Starks P.T. (2007). A Darwinian approach to Huntington’s disease: Subtle health benefits of a neurological disorder. Med. Hypoth..

[21] Gaba A.M., Zhang K., Marder K., Moskowitz C.B., Werner P., Boozer C.N. (2005). Energy balance in early-stage Huntington disease. Am. J. Clin. Nutr..

[22] Folstein S.E., Leigh R.J., Parhad I.M., Folstein M.F. (1986). The diagnosis of Huntington’s disease. Neurology.

[23] Johnson S.A., Stout J.C., Solomon A.C., Langbehn D.R., Aylward E.H., Cruce C.B., Ross C.A., Nance M., Kayson E., Julian-Baros E., Hayden M.R., Kieburtz K., Guttman M., Oakes D., Shoulson I., Beglinger L., Duff K., Penziner E., Paulsen J.S. (2007). Predict-HD Investigators of the Huntington Study Group. Beyond disgust: Impaired recognition of negative emotions prior to diagnosis in Huntington’s disease. Brain.

[24] Martin J.B., Gusella J.F. (1986). Huntington’s disease. Pathogenesis and management. N. Engl. J. Med..

[25] Morton A.J., Wood N.I., Hastings M.H., Hurelbrink C., Barker R.A., Maywood E.S. (2005). Disintegration of the sleep-wake cycle and circadian timing in Huntington’s disease. J. Neurosci..

[26] Phillips W., Shannon K.M., Barker R.A. (2008). The current clinical management of Huntington’s disease. Mov. Disord..

[27] Purdon S.E., Mohr E., Ilivitsky V., Jones B.D. (1994). Huntington’s disease: Pathogenesis, diagnosis and treatment. J. Psychiatry Neurosci..

[28] Roze E., Saudou F., Caboche J. (2008). Pathophysiology of Huntington’s disease: From huntingtin functions to potential treatments. Curr. Opin. Neurol..

[29] Sandyk R. (1992). Pineal and habenula calcification in schizophrenia. Int. J. Neurosci..

[30] Shiwach R.S., Norbury C.G. (1994). A controlled psychiatric study of individuals at risk for Huntington’s disease. Br. J. Psychiatry.

[31] Van Duijn E., Kingma E.M., van der Mast R.C. (2007). Psychopathology in verified Huntington’s disease gene carriers. J. Neuropsychiatry. Clin. Neurosci..

[32] Walker F. (2007). Huntington’s disease. Lancet.

[33] Cha J.H. (2000). Transcriptional dysregulation in Huntington’s disease. Trends Neurosci..

[34] La Spada A.R., Roling D.B., Harding A.E., Warner C.L., Spiegel R., Hausmanowa- Petrusewicz I., Yee W.C., Fischbeck K.H. (1992). Meiotic stability and genotype-phenotype correlation of the trinucleotide repeat in X-linked spinal and bulbar muscular atrophy. Nat. Genet..

[35] DiFiglia M., Sapp E., Chase K.O., Davies S.W., Bates G.P., Vonsattel J.P., Aronin N. (1997). Aggregation of huntingtin in neuronal intranuclear inclusions and dystrophic neurites in brain. Science.

[36] Rangone H., Humbert S., Saudou F. (2004). Huntington’s disease: How does huntingtin, an anti-apoptotic protein, become toxic?. Pathol. Biol. (Paris).

[37] Brouillet E., Condé F., Beal M.F., Hantraye P. (1999). Replicating Huntington’s disease phenotype in experimental animals. Prog. Neurobiol..

[38] Chiarugi A., Calvani M., Meli E., Traggiai E., Moroni F. Synthesis and release of neurotoxic kynurenine metabolites by human monocyte-derived macrophages. J. Neuroimmunol..

[39] Chiarugi A., Meli E., Moroni F. (77). Similarities and differences in the neural death processes activated by 3OH-Kynurenine and quinolinic acid. J. Neurochem..

[40] Estrada-Sánchez A.M., Mejía-Toiber J., Massieu L. (2008). Excitotoxic neuronal death and the pathogenesis of Huntington’s disease. Arch. Med. Res..

[41] Dawson R., Beal M.F., Bondy S.C., Di Monte D.A., Isom G.E. (1995). Excitotoxins, aging, and environmental neurotoxins: Implications for understanding human neurodegenerative diseases. Toxicol. Appl. Pharmacol..

[42] Guidetti P., Bates G.P., Graham R.K., Hayden M.R., Leavitt B.R., MacDonald M.E., Slow E.J., Wheeler V.C., Woodman B., Schwarcz R. (2006). Elevated brain 3-hydroxykynurenine and quinolinate levels in Huntington disease mice. Neurobiol. Dis..

[43] Pérez-de la Cruz V., Santamaría A. Integrative hypothesis for Huntington’s disease: A brief review of experimental evidence. Physiol. Res..

[44] Brouillet E., Jacquard C., Bizat N., Blum D. (2005). 3-Nitropropionic acid: A mitochondrial toxin to uncover physiopathological mechanisms underlying striatal degeneration in Huntington's disease. J. Neurochem..

[45] Brennan W.A., Bird E.D., Aprille J.R. (1985). Regional mitochondiral respieratoy activity in Huntington’s disease brain. J. Neurochem..

[46] Parker W.D., Baoyson S.J., Luder A.S., Parks J.K. (1990). Evidence for a defect in NADH: Ubiquinone oxidoreductase (complex I) in Huntington’s disease. Neurology.

[47] Arenas J., Campos Y., Ribacoba R., Martín M.A., Rubio J.C., Ablanedo P., Cabello A. (1998). Complex I defect in muscle from patients with Huntington’s disease. Ann. Neurol..

[48] Kumar P., Kumar A. (2009). Protective effect of rivastigmine against 3-nitropropionic acid-induced Huntington’s disease like symptoms: Possible behavioural, biochemical and cellular alterations. Eur. J. Pharmacol..

[49] Túnez I., Santamaría A. (2009). Model of Huntington’s disease induced with 3-nitropropionic acid. Rev. Neurol..

[50] Tasset I., Sánchez F., Túnez I. (2009). The molecular bases of Huntington’s disease: The role played by oxidative stress. Rev. Neurol..

[51] Gil J.M., Rego A.C. (2008). Mechanisms of neurodegeneration in Huntington’s disease. Eur. J. Neurosci..

[52] Beal M.F. (1995). Aging, energy, and oxidative stress in neurodegenerative disease. Ann. Neurol..

[53] Banoei M.M., Houshmand M., Panahi M.S., Shariati P., Rostami M., Manshadi M.D., Majidizadeh T. (2007). Huntington’s disease and mitochondrial DNA deletions: Event or regular mechanism for mutant huntingtin protein and CAG repeats expansion?!. Cell. Mol. Neurobiol..

[54] Lim D., Fedrizzi L., Tartari M., Zuccato C., Cattaneo E., Brini M., Carafoli E. (2008). Calcium homeostasis and mitochondrial dysfunction in striatal neurons of Huntington disease. J. Biol. Chem..

[55] Kalonia H., Kumar P., Kumar A., Nehru B. (2009). Effects of caffeic acid, rofecoxib, and their combination against quinolinic acid-induced behavioral alterations and disruption in glutathione redox status. Neurosci. Bull..

[56] Kumar P., Kalonia H., Kumar A. (2009). Lycopene modulates nitric oxide pathways against 3-nitropropioni acid-induced neurotoxicity. Life Sci..

[57] Kumar P., Kumar A. (2010). Protective effect of hesperidin and naringin against 3-nitropropionic acid induced Huntington’s like symptoms in rats: Possible role of nitric oxide. Behav. Brain Res..

[58] Browne S.E. (2008). Mitochondrial and Huntingon’s disease pathogenesis: Insight from genetic and chemical models. Ann. N.Y. Acad..

[59] Vonsattel J.P., Myers R.H., Stevens T.J., Ferrante R.J., Bird E.D., Richardson E.P. (1985). Neuropathological classification of Huntington’s disease. J. Neuropathol. Exp. Neurol..

[60] Bence N.F., Sampat R.M., Kopito R.R. (2001). Impairment of the ubiquitin-proteasome system by protein aggregation. Science.

[61] Finkbeiner S., Mitra S. (2008). The ubiquitin-proteasome pathway in Huntington’s disease. Sci.Word J..

[62] Gafni J., Hermel E., Young J.E., Wellington C.L., Hayden M.R., Ellerby L.M. (2004). Inhibition of calpain cleavage of huntingtin reduces toxicity: Accumulation of calpain/caspase fragments in the nucleus. J. Biol. Chem..

[63] Lunkes A., Lindenberg K.S., Ben-Haïem L., Weber C., Devys D., Landwehrmeyer G.B., Mandel J.L., Trottier Y. (2002). Proteases acting on mutant huntingtin generate cleaved products that differentially build up cytoplasmic and nuclear inclusions. Mol. Cell..

[64] Sánchez I., Mahlke C., Yuan J. (2003). Pivotal role of oligomerization in expanded polyglutamine neurodegenerative disorders. Nature.

[65] Poirier M.A., Li H., Macosko J., Cai S., Amzel M., Ross C.A. (2002). Huntingtin spheroids and protofibrils as precursors in polyglutamine fibrilization. J. Biol. Chem..

[66] Poirier M.A., Jiang H., Ross C.A. (2005). A structure-based analysis of huntingtin mutant polyglutamine aggregation and toxicity: Evidence for a compact beta-sheet structure. Hum. Mol. Genet..

[67] Ross C.A., Pickart C.M. (2004). The ubiquitin-proteasome pathway in Parkinson’s disease and other neurodegenerative diseases. Trends Cell Biol..

[68] Venkatraman P., Wetzel R., Tanaka M., Nukina N., Goldberg A.L. (2004). Eukaryotic proteasomes cannot digest polyglutamine sequences and release them during degradation of polyglutamine-containing proteins. Mol. Cell..

[69] Canals J.M., Pineda J.R., Torres-Peraza J.F., Bosch M., Martín-Ibáñez R., Muñoz M.T., Mengod G., Ernfors P., Alberch J. (2004). Brain-derived neurotrophic factor regulates the onset and severity of motor dysfunction associated with enkephalinergic neuronal degeneration in Huntington's disease. J. Neurosci..

[70] Cattaneo E. (2003). Dysfunction of wild-type huntingtin in Huntington disease. News Physiol. Sci..

[71] Jiang H., Poirier M.A., Liang Y., Pei Z., Weiskittel C.E., Smith W.W., DeFranco D.B., Ross C.A. (2006). Depletion of CBP is directly linked with cellular toxicity caused by mutant huntingtin. Neurobiol. Dis..

[72] Kegel K.B., Meloni A.R, Yi Y., Kim Y.J., Doyle E., Cuiffo B.G., Sapp E., Wang Y., Qin Z.H., Chen J.D., Nevins J.R., Aronin N., DiFiglia M. (2002). Huntingtin is present in the nucleus, interacts with the transcriptional corepressor C-terminal binding protein, and represses transcription. J. Biol. Chem..

[73] Lee S.T., Kim M. (2006). Aging and neurodegeneration. Molecular mechanisms of neuronal loss in Huntington’s disease. Mech Ageing Dev..

[74] Liu Y.F., Deth R.C., Devys D. (1997). SH3 domain-dependent association of huntingtin with epidermal growth factor receptor signalling complexes. J. Biol. Chem..

[75] Martindale D., Hackam A., Wieczorek A., Ellerby L., Wellington C., McCutcheon K., Singaraja R., Kazemi-Esfarjani P., Devon R., Kim S.U., Bredesen D.E., Tufaro F.M , Hayden M.R. (1998). Length of huntingtin and its polyglutamine tract influences localization and frequency of intracellular aggregates. Nat. Genet..

[76] Ross C.A., Poirier M.A. (2004). Protein aggregation and neurodegenerative disease. Nat. Med..

[77] Scappini E., Koh T.W., Martin N.P., O’Bryan J.P. (2007). Intersectin enhances huntingtin aggregation and neurodegeneration through activation of c-Jun-NH2-terminal kinase. Hum. Mol. Genet..

[78] Steffan J.S., Agrawal N., Pallos J., Rockabrand E., Trotman L.C., Slepko N., Illes K, Lukacsovich T., Zhu Y.Z., Cattaneo E., Pandolfi P.P., Thompson L.M., Marsh J.L. (2004). SUMO modification of Huntingtin and Huntington’s disease pathology. Science.

[79] Sugars K.L., Rubinsztein D.C. (2003). Transcriptional abnormalities in Huntington disease. Trends Genet..

[80] Zuccato C., Liber D., Ramos C., Tarditi A., Rigamonti D., Tartari M., Valenza M., Cattaneo E. (2003). Progressive loss of BDNF in a mouse model of Huntington’s disease and rescue by BDNF delivery. Pharmacol. Res..

[81] Coyle J.T., Schwarcz R. (1976). Lesion of striatal neurones with kainic acid provides a model for Huntington’s chorea. Nature.

[82] Rothman S.M., Olney J.W. (1995). Excitotoxicity and the NMDA receptor-still lethal after eight years. Trends Neurosci..

[83] Mangiarini L., Sathasivam K., Seller M., Cozens B., Harper A., Hetherington C., Lawton M., Trottier Y., Lehrach H., Davies S.W., Bates G.P. (1996). Exon 1 of the HD gene with an expanded CAG repeat is sufficient to cause a progressive neurological phenotype in transgenic mice. Cell.

[84] Parker J.A., Connolly J.B., Wellington C., Hayden M., Dausset J., Neri C. (2001). Expanded poluglutamines in Caenorhabditis elegans cause axonal abnormalities and severe dysfunction of PLM mechanosensory neurons without cell death. Proc. Natl. Acad. Sci. USA.

[85] Marsh J.L., Pallos J., Thompson L.M. (2003). Fly models of Huntington’s disease. Hum. Mol. Genet..

[86] Powers W.J., Videen T.O., Markham J., McGee-Minnich L., Antenor-Dorsey J.V., Hershey T., Perlmutter J.S. (2007). Selective defect of *in vivo* glycolysis in early Huntington’s disease striatum. Proc. Natl. Acad. Sci. USA.

[87] Beal M.F. (2007). Does impairment of energy metabolism result in excitotoxic neuronal death in neurodegenerative illnesses?. Ann. Neurol..

[88] Hamilton B.F., Gould D.H. (1987). Nature and distribution of brain lesions in rats intoxicated with 3-nitropropionic acid: A type of hypoxic (energy deficient) brain damage. Acta Neuropathol..

[89] Liu X., Luo X., Hu W. (1992). Studies on the epidemiology and etiology of moldy sugarcane poisoning in China. Biomed. Environ. Sci..

[90] Ramaswamy S., McBrid J.L., Kordower J.H. (2007). Animal models of Huntington’s disease. ILAR. J..

[91] Borlongan C.V., Koutouzis T.K., Freeman T.B., Hauser R.A., Cahill D.W., Sanberg R. Hyperactivity and hypoactivity in a rat model of Huntington’s disease: The systemic 3-nitropropionic acid model. Brain Res. Brain Res. Protoc..

[92] Ouary S., Bizat N., Altairac S., Ménétrat H., Mittoux V., Condé F., Hantraye P., Brouillet E. (2000). Major strain differences in response to chronic systemic administration of the mitochondrial toxin 3-nitropropionic acid in rats: Implications for neuroprotection studies. Neuroscience.

[93] Ahuja M., Bishnoi M., Chopra K. (2008). Protective effect of minocycline, a semi-synthetic second-generation tetracycline against 3-nitropropionic acid (3-NP)-induced neurotoxicity. Toxicology.

[94] Deshpande S.B., Hida H., Takei-Io N., Masuda T., Baba H., Nishino H. (2006). Involvement of nitric oxide in 3-nitropropionic acid-induced striatal toxicity in rats. Brain Res..

[95] Dhir A., Akula KK., Kulkarni S.K., Tiagabine A. (2008). GABA uptake inhibitor, attenuates 3-nitropropionic acid-induced alterations in various behavioral and biochemical parameters in rats. Prog. Neuropsychopharmacol. Biol. Psychiatry.

[96] Lukács A., Szabó A., Vezér T., Papp A. (2006). The acute effects of 3-nitropropionic acid on the behavior and spontaneous cortical electrical activity of rats. Acta Neurobiol. Exp. (Wars)..

[97] McBride J.L., During M.J., Wuu J., Chen E.Y., Leurgans S.E., Kordower J.H. (2003). Structural and functional neuroprotection in a rat model of Huntington`s disease by viral gene transfer of GDNF. Exp. Neurol..

[98] Mogami M., Hayashi Y., Masuda T., Kohri K., Nishino H., Hida H. (2008). Altered striatal vulnerability to 3-nitropropionic acid in rats due to sex hormone levels during late phase of brain development. Neurosci. Lett..

[99] Przybyla-Zawislak B.D., Thorn B.T., Ali S.F., Dennis R.A., Amato A., Virmani A., Binienda Z.K. (2006). Identification of rat hippocampal mRNAs altered by the mitochondrial toxicant, 3-NPA. Ann. N. Y. Acad. Sci..

[100] Túnez I., Collado J.A., Medina F.J., Peña J., Muñoz M.C., Jimena I., Franco F., Rueda I., Feijóo M., Muntané J., Montilla P. 17 Beta-estradiol may affect vulnerability of striatum in a 3-nitropropionic acid-induced experimental model of Huntington's disease in ovariectomized rats. Neurochem. Int..

[101] Túnez I., Drucker-Colín R., Jimena I., Medina F.J., Muñoz M.C., Peña J., Montilla P. Transcranial magnetic stimulation attenuates cell loss and oxidative damage in the striatum induced in the 3-nitropropionic model of Huntington's disease. J. Neurochem..

[102] Túnez I., Feijóo M., Collado J.A., Medina F.J., Peña J., Muñoz M.C., Jimena I., Franco F., Rueda I., Muntané J., Montilla P. (2007). Effect of testosterone on oxidative stress and cell damage induced by 3-nitropropionic acid in striatum of ovariectomized rats. Life Sci..

[103] Túnez I., Montilla P., Muñoz M.C., Feijóo M., Salcedo M. Protective effect of melatonin on 3-nitropropionic acid-induced oxidative stress in synaptosomes in an animal model of Huntington’s disease. J. Pineal Res..

[104] Túnez I., Montilla P., Muñoz M.C., Drucker-Colín R. Effect of nicotine on 3-nitropropionic acid-induced oxidative stress in synaptosomes. Eur. J. Pharmacol..

[105] Túnez I., Muñoz M.C., Montilla P. (2005). Treatment with dehydroepiandrosterone prevents oxidative stress induced by 3-nitropropionic acid in synaptosomes. Pharmacology.

[106] Shih A.Y., Imbeault S., Barakauskas V., Erb H., Jiang L., Li P., Murphy T.H. (2005). Induction of the Nrf2-driven antioxidant response confers neuroprotection during mitochondrial stress *in vivo*. J. Biol. Chem..

[107] Borlongan C.V., Koutouzis T.K., Sanberg P.R. (1997). 3-Nitropropionic acid animal model and Huntington’s disease. Neurosci. Biobehav. Rev..

[108] Beal M.F., Brouillet E., Jenkins B.G., Ferrante R.J., Kowall N.W., Miller J.M., Storey E., Srivasta R., Rosen B.R., Hyman B.T. (1993). Neurochemical and histologic characterization of striatal excitotoxic lesions produced by the mitochondrial toxin 3-ntropropionic acid. J. Neurosci..

[109] Guyot M.C., Hantraye P., Dolan R., Palfi S., Maziére M., Brouillet E. (1997). Quantifiable bradykinesia, gait abnormalities and Huntington's disease-like striatal lesions in rats chronically treated with 3-nitropropionic acid. Neuroscience.

[110] Hamilton B.F., Gould D.H. (1987). Nature and distribution of brain lesions in rats intoxicated with 3-nitropropionic acid: A type of hypoxic (energy deficient) brain damage. Acta Neuropathol..

[111] Beal M.F. (1994). Neurochemistry and toxin models in Huntington’s disease. Curr. Opin. Neurol..

[112] Brouillet E., Guyot M.C., Mittoux V., Altairac S., Conde F., Palfi S., Hantraye P. (1998). Partial inhibition of brain succinate dehydrogenase by 3-nitropropionic acid is sufficient to initiate striatal degeneration in rat. J. Neurochem..

[113] Borlongan C.V., Koutouzis T.K., Freeman T.B., Cahill D.W., Sanberg P.R. Behavioral pathology induced by repeated systemic injections of 3-nitropropionic acid mimics the motoric symptoms of Huntington’s disease. Brain Res..

[114] Borlongan C.V., Koutouzis T.K., Randall T.S., Freeman T.B., Cahill D.W., Sanberg P.R. (1995). Systemic 3-nitropropionic acid: Behavioral deficits and striatal damage in adult rats. Brain Res. Bull..

[115] Brouillet E., Hantraye P., Ferrante R.J., Dolan R., Leroy-Willig A., Kowall N.W., Beal M.F. (1995). Chronic mitochondrial energy impairment produces selective striatal degeneration and abnormal choreiform movements in primates. Proc. Natl. Acad. Sci. USA.

[116] Mettler F.A. (1972). Choreoathetosis and striopallidonigral necrosis due to sodium azide. Exp. Neurol..

[117] Palfi S., Ferrante R.J., Brouillet E., Beal M.F., Dolan R., Guyot M.C., Peschanski M., Hantraye P. (1996). Chronic 3-nitropropionic acid treatment in baboons replicates the cognitive and motor deficits of Huntington’s disease. J. Neurosci..

[118] Stober T., Wussow W., Schimrigk K. (1984). Bicaudate diameter the most specific and simple CT parameter in the diagnosis of Huntington’s disease. Neuroradiology.

[119] Ming L. (1995). Moldy sugarcane poisoning-a case report with a brief review. J. Toxicol. Clin. Toxicol..

[120] Ludolph A.C., He F., Spencer P.S., Hammerstad J., Sabri M. (1991). 3-Nitropropinic acid-exogenous animal neurotoxin and possible human striatal toxin. Can. J. Neurol. Sci..

[121] Alston T.A., Mela L., Bright H.J. (1977). Nitropropionate, the toxic substance of Indigofera, is a suicide inactivator of succinate dehydrogenase. Proc. Natl. Acad. Sci. USA.

[122] Browne S.E., Bowling A.C., MacGarvey U., Baik M.J., Berger S.C., Muqit M.M., Bird E.D., Beal M. (1997). Oxidative damage and metabolic dysfunction in Huntington's disease: Selective vulnerability of the basal ganglia. Ann. Neurol..

[123] Gu M., Gash M.T., Mann V.M., Javoy-Agid F., Cooper J.M., Schapira A.H. (199). Mitochondrial defect in Huntington’s disease caudate nucleus. Ann. Neurol..

[124] Tabrizi S.J., Cleeter M.W., Xuereb J., Taanman J.W., Cooper J.M., Schapira A.H. (1999). Biochemical abnormalities and excitotoxicity in Huntington’s disease brain. Ann. Neurol..

[125] Lee J.M., Shih A.Y., Murphy T.H., Johnson J.A. (2003). NF-E2-related factor-2 mediates neuroprotection against mitochondrial complex I inhibitors and increased concentrations of intracellular calcium in primary cortical neurons. J. Biol. Chem..

[126] Nasr P., Gursahani H.I., Pang Z., Bondada V., Lee J., Hadley R.W., Geddes J.W. (2003). Influence of cytosolic and mitochondrial Ca^2+^, ATP, mitochondrial membrane potential, and calpain activity on the mechanism on neuron death induced by 3-nitropropionic acid. Neurochem. Int..

[127] Maciel E.N., Kowaltowski A.J., Schwalm F.D., Rodrigues J.M., Souza D.O., Vercesi A.E., Wajner M., Castilho R.F. (2004). Mitochondrial permeability transition in neuronal damage promoted by Ca^2+^ and respiratory chain complex II inhibition. J. Neurochem..

[128] Galas M.C., Bizat N., Cuvelier L., Bantubungi K., Brouillet E., Schiffmann S.N., Blum D. (2004). Death of cortical and striatal neurons induced by mitochondrial defect involves differential molecular mechanisms. Neurol. Dis..

[129] Alexi T., Hughes P.E., Faull R.L., Williams C.E. (1998). 3-Nitropropionic acid’s lethal triplet: Cooperative pathways of neurodegeneration. NeuroReport.

[130] Deshpande S.B., Fukuda A., Nishino H. (1997). 3-Nitropropionic acid increases the intracellular Ca^2+^ in cultured astrocytes by reverse operation of the Na^+^-Ca^2+^ exchanger. Exp. Neurol..

[131] Fukuda A., Deshpande S.B., Shimano Y., Nishino H. (1998). Astrocytes are more vulnerable than neurons to cellular Ca^2+^ overload induced by a mitochondrial toxin, 3-nitropropionic acid. Neuroscience.

[132] Montilla P., Túnez I., Muñoz M.C., Salcedo M., Feijóo M., Muñoz-Castañeda J.R., Bujalance I. (2004). Effect of glucocorticoids on 3-nitropropionic acid-induced oxidative stress in synaptosomes. Eur. J. Pharmacol..

[133] Lafon-Cazal M., Pietri S., Culcasi M., Bockaert J. (1993). NMDA-dependent superoxide production and neurotoxicity. Nature.

[134] La Fontaine M.A., Geddes J.W., Banks A., Butterfield D.A. (2000). 3-Nitropropionic acid induced *in vivo* protein oxidation in striatal and cortical synaptosomes: Insights into Huntington`s disease. Brain Res..

[135] Marti M., Mela F., Ulazzi L., Hanau S., Stocchi S., Paganini F., Beani L., Bianchi C., Morari M. (2003). Differential responsiveness of rat striatal nerve endings to the mitochondrial toxin 3-nitropropionic acid: Implications for Huntington’s disease. Eur. J. Neurosci..

[136] Storgaard J., Kornblit B.T., Zimmer J., Gramsbergen J.B. (2000). 3-Nitropropionic acid neurotoxicity in organotypic striatal and corticostriatal slice cultures is dependent on glucose and glutamate. Exp. Neurol..

[137] Novelli A., Reilly A., Lysk P.G., Henneberry R.C. (1988). Glutamate becomes neurotoxic via the N-methyl-D-aspartate receptor when intracellular energy levels are reduced. Brain Res..

[138] Beal M.F., Kowall N.W., Ellison D.W., Mazurek M.F., Swartz K.J., Martin J.B. (1986). Replication of the neurochemical characteristics of Huntington’s disease by quinolinic acid. Nature.

[139] Nasr P., Carbery T., Geddes J.W. (2009). N-methyl-D-aspartate receptor antagonists have variable affect in 3-nitropropionic acid toxicity. Neurochem. Res..

[140] Moro M.A., De Alba J., Cárdenas A., De Cristóbal J., Leza J.C., Lizasoain I.M., Díaz-Guerra M.J., Boscá L., Lorenzo P. (2000). Mechanisms of the neuroprotective effect of aspirin after oxygen and glucose deprivation in rat forebrain slices. Neuropharmacology.

[141] Ryu J.K., Nagai A., Kim J., Lee M.C., McLarnon J.G., Kim S.U. (2003). Microglial activation and cell death induced by the mitochondial toxin 3-nitropropionic acid: *In vitro* and *in vivo* studies. Neurobiol. Dis..

[142] Ohgoh M., Shimizu H., Ogura H., Nishizawa Y. (2000). Astroglial trophic support and neuronal cell death: influence of cellular energy level on type of cell death induced by mitochondrial toxin in cultured rat cortical neurons. J. Neurochem..

[143] Mittoux V., Ouary S., Monville C., Lisovoski F., Poyot T., Conde F., Escartin C., Robichon R., Brouillet E., Peschanski M., Hantraye P. (2002). Corticostriatopallidal neuroprotection by adenovirus-mediated ciliary neurotrophic factor gene transfer in a rat model of progressive striatal degeneration. J. Neurosci..

[144] Villarán R.F., Tomás-Camardiel M., de Pablos R.M., Santiago M., Herrera A.J., Navarro A., Machado A., Cano J. (2008). Endogenous dopamine enhances the neurotoxicity of 3-nitropropionic acid in the striatum through the increase of mitochondrial respiratory inhibition and free radicals production. Neurotoxicology.

[145] Nishino H., Kumazaki M., Fukuda A., Fujimoto I., Shimano Y., Hida H., Sakurai T., Deshpande S.B., Shimizu H., Morikawa S., Inubushi T. (1997). Acute 3-nitropropionic acid intoxication induces striatal astrocytic cell death and dysfunction of the blood-brain barrier: Involvement of dopamine toxicity. Neurosci. Res..

[146] McCracken E., Dewar D., Hunter A.J. (2001). White matter damage following systemic injection of the mitochondrial inhibitor 3-nitropropionic acid in rat. Brain Res..

[147] Sato S., Gobbel G.T., Honkaniemi J., Li Y., Kondo T., Murakami K., Sato M., Copin J.C., Chan P.H. (1997). Apoptosis in the striatum of rats following intraperitoneal injection of 3-nitropropionic acid. Brain Res..

[148] Sato S., Gobbel G.T. (1997). Blood-brain barrier disruption, HSP70 expression and apoptosis due to 3-nitropropionic acid, a mitochondrial toxin. Acta Neurochir. Suppl..

[149] Bantubungi K., Jacquard C., Greco A., Pintor A., Chtarto A., Tai K., Galas M.C., Tenenbaum L., Déglon N., Popoli P., Minghetti L., Brouillet E., Brtchi J., Levivier M., Schiffmann S.N., Blum D. (2005). Minocycline in phenotypic models of Huntington’s disease. Neurobiol. Dis..

[150] Nishino H., Hida H., Kumazaki M., Shimano Y., Nakajima K., Shimizu H., Ooiwa T., Baba H. (2000). The striatum is the most vulnerable region in the brain to mitochondrial energy compromise: A hypothesis to explain its specific vulnerability. J. Neurotrauma.

[151] Halliwell B. (2006). Oxidative stress and neurodegeneration: Where are we now?. J. Neurochem..

[152] Moncada S., Bolaños J.P. (2006). Nitric oxide, cell bioenergetics and neurodegeneration. J. Neurochem..

[153] Coles C.J., Edmondson D.E., Singer T.P. (1979). Inactivation of succinate dehydrogenase by 3-nitropropionate. J. Biol. Chem..

[154] Beal M.F., Hyman B.T., Koroshetz W. (1993). Do defects in mitochondrial energy metabolism underlie the pathology of neurodegenerative diseases?. Trends Neurosci..

[155] Palfi S., Leventhal L., Goetz C.G., Hantraye T., Roitberg B.Z., Sramek J., Emborg M., Kordower J.H. (2000). Delayed onset of progressive dystonia following subacute 3-nitropropionic acid treatment in Cebus apella monkeys. Mov. Disord..

[156] Yang L., Calingasan N.Y., Chen J., Ley J.J., Becker D.A., Beal M.F. (2005). A novel azulenyl nitrone antioxidant protects against MPTP and 3-nitropropionic acid neurotoxicities. Exp. Neurol..

[157] Binienda Z., Simmons C., Hussain S., Slikker W., Ali S.F. (1998). Effect of acute exposure to 3-nitropropionic acid on activities of endogenous antioxidants in the rat brain. Neurosci. Lett..

[158] Herrera-Mundo M.N., Silva-Adaya D., Maldonado P.D., Galván-Arzate S., Andrés-Martínez L., Pérez-de la Cruz V., Pedraza-Chaverrí J., Santamaría A. (2006). S-Allyslcysteine prevents the rat from 3-nitropropionic acid-induced hypeactivity, early markers of oxidative stress and mitochondrial dysfunction. Neurosci. Res..

[159] Nam E., Lee S.M., Koh S.E., Joo W.S., Maeng S., Im H.I., Kim Y.S. (2005). Melatonin protects against neuronal damage induced by 3-nitropropionic acid in rat striatum. Brain Res..

[160] Pérez-de la Cruz V., González-Cortés C., Pedraza-Chaverrí J., Maldonado P.D., Andrés-Martínez L., Santamaría A. (2006). Protective effect of S-allylcysteine on 3-nitropropionic acid-induced lipid peroxidation and mitochondrial dysfunction in rat brain synaptosomes. Brain Res. Bull..

[161] Schulz J.B., Henshaw D.R., MacGarvey U., Beal M.F. (1996). Involvement of oxidative stress in 3-nitropropionic acid neurotoxicity. Neurochem. Int..

[162] Pérez-de la Cruz V., Konigsberg M., Pedraza-Chaverri J., Herrera-Mundo N., Díaz-Muñoz M., Morán J., Fortoul-van der Goes T., Rondán-Zárate A., Maldonado P.D., Ali S.F., Santamaría A. (2008). Cytoplasmic calcium mediates oxidative damage in an excitotoxic/energetic deficit synergic model in rats. Eur. J. Neurosci..

[163] Ferdinandy P., Schulz R. (2003). Nitric oxide, superoxide, and peroxynitrite in myocardial ischaemia-reperfusion injury and preconditioning. Br. J. Pharmacol..

[164] Blum D., Gall D., Cuvelier L., Schiffmann S.N. (2001). Topological analysis of striatal lesions induced by 3-nitropropionic acid in the Lewis rat. Neuroreport.

[165] Pang Z., Geddes J.W. (1997). Mechanisms of cell death induced by the mitochondrial toxin 3-nitropropionic acid: Acute excitotoxic necrosis and delayed apoptosis. J. Neurosci..

[166] Bossi S.R., Simpson J.R., Isacson O. (1993). Age dependence of striatal neuronal death caused by mitochondrial dysfunction. Neuroreport.

[167] Koutouzis T.K., Borlongan C.V., Scorcia T., Creese I., Cahill D.W., Freeman T.B., Sanberg P.R. (1994). Systemic 3-nitropropionic acid: Long-term effects on locomotor behavior. Brain Res..

[168] Filloux F., Townsend J.J. (1993). Pre- and postsynaptic neurotoxic effects of dopamine demostrated by intrastriatal injection. Exp. Neuro..

[169] Reynolds D.S., Carter R.J., Morton A.J. (1998). Dopamine modulates the susceptibility of striatal neurons to 3-nitropropionic acid in the rat model of Huntington`s disease. J. Neurosci..

[170] Maragos W.F., Young K.L., Altman C.S., Pocernich C.B., Drake J., Butterfield D.A., Seif I., Holschneider D.P., Chen K., Shih J.C. (2004). Striatal damage and oxidative stress induced by the mitochondrial toxin malonate are reduced in clorgyline-treated rats and MAO-A deficient mice. Neurochem. Res..

[171] Blum D., Hourez R., Galas M.C., Popoli P., Schiffmann S.N. (2003). Adenosine receptors and Huntington’s disease: Implications for pathogenesis and therapeutics. Lancet Neurol..

[172] Lastres-Becker I., Fezza F., Cebeira M., Bisogno T., Ramos J.A., Milone A., Fernández-Ruiz J., Di Marzo V. (2001). Changes in endocannabinoid transmission in the basal ganglia in a rat model of Huntington’s disease. Neuroreport.

[173] Lastres-Becker I., Gómez M., De Miguel R., Ramos J.A., Fernández-Ruiz J. (2002). Loss of Cannabinoid CB(1) receptors in the basal ganglia in the late akinetic phase of rats with experimental Huntington’s disease. Neurotox Res..

[174] Canals J.M., Checa N., Marco S., Akerud P., Michels A., Pérez-Navarro E., Tolosa E., Arenas E., Alberch J. (2001). Expression of brain-derived neurotrophic factor in cortical neurons is regulated by striatal target area. J. Neurosci..

[175] Gauthier L.R., Charrin B.C., Borrell-Pagès M., Dompierre J.P., Rangone H., Cordelières F.P., De Mey J., MacDonald M.E., Lessmann V., Humbertm S., Saudou F. (2004). Huntingtin controls neurotrophic support and survival of neurons by enhancing BDNF vesicular transport along microtubules. Cell.

[176] Zuccato C., Ciammola A., Rigamonti D., Leavitt B.R., Goffredo D., Conti L., MacDonald M.E, Friedlander R.M., Silani V., Hayden M.R., Timmusk T.Ç., Sipione S., Cattaneo E. (2001). Loss of huntingtin-mediated BDNF gene transcription in Huntington’s disease. Science.

[177] Zuccato C., Tartari M., Crotti A., Goffredo D., Valenza M., Conti L., Cataudella T., Leavitt B.R., Hayden M.R., Timmusk T., Rigamonti D., Cattaneo E. (2003). Huntingtin interacts with REST/NRSF to modulate the transcription of NRSE-controlled neuronal genes. Nat. Genet..

[178] Zuccato C., Cattaneo E. (2007). Role of brain-derived neurotrophic factor in Huntington's disease. Prog. Neurobiol..

[179] Altar C.A., Cai N., Bliven T., Juhasz M., Conner J.M., Acheson A.L., Lindsay R.M., Wiegand S.J. (1997). Anterograde transport of brain-derived neurotrophic factor and its role in the brain. Nature.

[180] Kovalchuk Y., Hanse E., Kafitz KW., Konnerth A. (2002). Postsynaptic induction of BDNF-mediated long-term potentiation. Science.

[181] Ferrer I., Goutan E., Marín C., Rey M.J., Ribalta T. (2000). Brain-derived neurotrophic factor in Huntington disease. Brain Res..

[182] Ernfors P., Bramham C.R. (2003). The coupling of a trkB tyrosine residue to LTP. Trends Neurosci..

[183] Almli C.R., Levy T.J., Han B.H., Shah A.R., Gidday J.M., Holtzman D.M. (2000). BDNF protects against spatial memory deficits following neonatal hypoxia-ischemia. Exp. Neurol..

[184] Hock C., Heese K., Hulette C., Rosenberg C., Otten U. (2000). Region-specific neurotrophin imbalances in Alzheimer disease: Decreased levels of brain-derived neurotrophic factor and increased levels of nerve growth factor in hippocampus and cortical areas. Arch. Neurol..

[185] Hellweg R., von Arnim C.A., Büchner M., Huber R., Riepe M.W. (2003). Neuroprotection and neuronal dysfunction upon repetitive inhibition of oxidative phosphorylation. Exp. Neurol..

[186] Cho H.Y., Jedlicka A.E., Reddy S.P., Kensler T.W., Yamamoto M., Zhang L.Y., Kleeberger S.R. (2002). Role of NRF2 in protection against hyperoxic lung injury in mice. Am. J. Respir. Cell Mol. Biol..

[187] Gao X., Talalay P. (2004). Induction of phase 2 genes by sulforaphane protects retinal pigment epithelial cells against photooxidative damage. Proc. Natl. Acad. Sci. USA.

[188] Calkins M.J., Jakel R.J., Johnson D.A., Chan K., Kan Y.W., Johnson J.A. (2005). Protection from mitochondrial complex II inhibition *in vitro* and *in vivo* by Nrf2-mediated transcription. Proc. Natl. Acad. Sci. USA.

[189] Cullinan S.B., Diehl J.A. (2004). PERK-dependent activation of Nrf2 contributes to redox homeostasis and cell survival following endoplasmic reticulum stress. J. Biol. Chem..

[190] Cullinan S.B., Zhang D., Hannink M., Arvisais E., Kaufman R.J., Diehl J.A. (2003). Nrf2 is a direct PERK substrate and effector of PERK-dependent cell survival. Mol. Cell Biol..

[191] Kotlo K.U., Yehiely F., Harasty H., Hesabi B., Shchors K., Einat P., Rozen A., Berent E., Deiss L.P. (2003). Nrf2 is an inhibitor of the Fas pathway as identified by Achilles`Hell Method, a new function-based approach to gene identification in human cells. Oncogene.

[192] Almeida S., Brett A.C., Góis I.N., Oliveira C.R., Rego A.C. (2006). Caspase-dependent and **-**independent cell death induced by 3-nitropropionic acid in rat cortical neurons. J. Cell Biochem..

[193] Almeida S., Domingues A., Rodrigues L., Oliveira C.R., Rego A.C. (2004). FK506 prevents mitochondrial-dependent apoptotic cell death induced by 3-nitropropionic acid in rat primary cortical cultures. Neurobiol. Dis..

[194] Enoksson M., Robertson J.D., Gogvadze V., Bu P., Kropotov A., Zhivotovsky B., Orrenius S. (2004). Caspase-2 permeabilizes the outer mitochondrial membrane and disrupts the binding of cytochrome c to anionic phospholipids. J. Biol. Chem..

[195] Garcia M., Vanhoutte P., Pages C., Besson M.J., Brouillet E., Caboche J. (2002). The mitochondrial toxin 3-nitropropionic acid induces striatal neurodegeneration *via* a c-jun N-terminal kinase/c-Jun module. J. Neurosci..

[196] Ichijo H., Nishida E., Irie K., ten Dijke P., Saitoh M., Moriguchi T., Takagi M., Matsumoto K., Miyazono K., Gotoh Y. (1997). Induction of apoptosis by ASK1, a mammalian MAPKKK that activates SAPK/JNK and p38 signaling pathways. Science.

[197] Minn Y., Cho K.J., Kim H.W., Kim H.J., Suk S.H., Lee B.I., Kim G.W. (2008). Induction of apoptosis signal-regulating kinase 1 and oxidative stress mediate age dependent vulnerability to 3-nitropropionic acid in the mouse striatum. Neurosci. Lett..

[198] Pelegrí C., Duran-Vilaregut J., del Valle J., Crespo-Biel N., Ferrer I., Pallàs M., Camins A., Vilaplana J. (2008). Cell cycle activation in striatal neurons from Huntington’s disease patients and rats treated with 3-nitropropionic acid. Int. J. Dev. Neurosci..

[199] Höglinger G.U., Breunig J.J., Depboylu C., Rouaux C., Michel P.P., Alvarez-Fischer D., Boutillier A.L., Degregori J., Oertel W.H., Rakic P., Hirsch E.C., Hunot S. (2007). The pRb/E2F cell-cycle pathway mediates cell death in Parkinson’s disease. Proc. Natl. Acad. Sci. USA..

[200] Crespo-Biel N., Camins A., Pelegrí C., Vilaplana J., Pallàs M., Canudas A.M. (2007). 3-Nitropropionic acid activates calpain/cdk5 pathway in rat striatum. Neurosci. Lett..

[201] Park D.S., Obeidat A., Giovanni A., Greene L.A. (2000). Cell cycle regulators in neuronal death evoked by excitotoxic stress: Implications for neurodegeneration and its treatment. Neurobiol. Aging.

[202] Seidel B., Jiang L., Wolf G. (2000). Differentially displayed genes in neuroblastoma cells treated with a mitochondrial toxin: Evidence for possible involvement of ICAM-1 in 3-nitropropionic acid-mediated neurodegeneration. Toxicol. Lett..

[203] Napolitano M., Zei D., Centonze D., Palermo R., Bernardi G., Vacca A., Calabresi P., Gulino A. (2008). NF-kB/NOS cross-talk induced by mitochondrial complex II inhibition: Implications for Huntington`s disease. Neurosci. Lett..

[204] García-Ramos R., del Val-Fernández J., Catalán-Alonso M.J., Barcia-Albacar J.A., Matías-Guiu J. (2007). Experimental models of Huntington`s disease. Rev. Neurol..

[205] Qin Z.H., Wang J., Gu Z.L. (2005). Development of novel therapies for Huntington’s disease: Hope and challenge. Acta Pharmacol. Sin..

[206] Wang L.H., Qin Z.H. (2006). Animal models of Huntington`s disease: Implications in uncovering pathogenic mechanisms and developing therapies. Acta Pharmacol. Sin..

[207] Leavitt B.R., van Raamsdonk J.M., Shehadeh J., Fernandes H., Murphy Z., Graham R.K., Wellington C.L., Raymond L.A., Hayden M.R. (2006). Wild-type huntingtin protects neurons from excitotoxicity. J. Neurochem..

[208] Sanberg P.R., Coyle J.T. (1984). Scientific approaches to Huntington’s disease. CRC Crit. Rev. Clin. Neurobiol..

[209] Santamaría A., Jiménez M.E. (2006). Oxidative/nitrative stress, a common factor in different neurotoxic paradigms: An overview. Curr. Topics Neurochem..

[210] Schwarcz R., Foster A.C., French E.D., Whetsell W.O., Köhler C. (1984). Excitotoxic models for neurodegenerative disorders. Life Sci..

[211] Isacson O., Brundin P., Gage F.H., Björklund A. (1985). Neural grafting in a rat model of Huntington’s disease: Progressive neurochemical changes after neostriatal ibotenate lesions and striatal tissue grafting. Neuroscience.

[212] Hantraye P., Riche D., Maziere M., Isacson O. (1990). A primate model of Huntington’s disease: Behavioral and anatomical studies of unilateral excitotoxic lesions of the caudate-putamen in the baboon. Exp. Neurol..

[213] Pérez-de la Cruz V., Königsberg M., Santamaría A. Kynurenine pathway and disease: An overview. CNS Neurol. Disord. Drug Targets.

[214] Dang Y., Dale W.E., Brown O.R. (2000). Comparative effects of oxygen on indoleamine 2,3-dioxygenase and tryptophan 2,3-dioxygenase of the kynurenine pathway. Free Radic. Biol. Med..

[215] Guidetti P., Luthi-Carter R.E., Augood S.J., Schwarcz R. (2004). Neostriatal and cortical quinolinate levels are increased in early grade Huntington’s disease. Neurobiol. Dis..

[216] González-Cortés C., Santamaría A., Santamaría A., Jiménez-Capdeville M.E. (2007). New Perspectives on Brain Cell Damage, Neurodegeneration and Neuroprotective Strategies. Research Signpost.

[217] Santamaría A., Jiménez-Capdeville M.E., Camacho A., Rodríguez-Martínez E., Flores A., Galván-Arzate S. (2001). *In vivo* hydroxyl radical formation after quinolinic acid infusion into rat corpus striatum. Neuroreport.

[218] Stone T.W. (1993). Neuropharmacology of quinolinic and kynurenic acids. Pharmacol. Rev..

[219] Heyes M.P., Lackner A. (1990). Increased cerebrospinal fluid quinolinic acid, kynurenic acid, and L-kynurenine in acute septicemia. J. Neurochem..

[220] Heyes M.P., Mefford I.N., Quearry B.J., Dedhia M., Lackner A. (1990). Increased ratio of quinolinic acid to kynurenic acid in cerebrospinal fluid of D retrovirus-infected rhesus macaques: Relationship to clinical and viral status. Ann. Neurol..

[221] Heyes MP., Saito K., Crowley JS., Davis LE., Demitrack M.A., Der M., Dilling L.A., Elia J., Kruesi M.J., Lackner A., Larsen SA., Lee K., Leonard HL., Markey SP., Martin A., Milstein S., Mouradian MM., Pranzatelli MR., Quearry BJ., Salazar A., Smith M., Strauss SE., Sunderland T., Swedo SW., Tourtellotte WW. (1992). Quinolinic acid and kynurenine pathway metabolism in inflammatory and non-inflammatory neurological disease. Brain.

[222] Moroni F., Lombardi G., Carlà V., Pellegrini D., Carassale G.L., Cortesini C. (1986). Content of quinolinic acid and of other tryptophan metabolites increases in brain regions of rats used as experimental models of hepatic encephalopathy. J. Neurochem..

[223] Ogawa T., Matson W.R., Beal M.F., Myers R.H., Bird E.D., Milbury P., Saso S. (1992). Kynurenine pathway abnormalities in Parkinson’s disease. Neurology.

[224] Schwarcz R., Okuno E., White R.J., Bird E.D., Whetsell W.O. (1988). 3-Hydroxyanthranilate oxygenase activity is increased in the brains of Huntington disease victims. Proc. Natl. Acad. Sci. USA.

[225] Stone T.W. (2001). Endogenous neurotoxins from tryptophan. Toxicology.

[226] Stone T.W., Connick J.H. (1985). Quinolinic acid and other kynurenines in the central nervous system. Neuroscience.

[227] During M.J., Heyes M.P., Freese A., Markey S.P., Martin J.B., Roth R.H. (1989). Quinolinic acid concentrations in striatal extracellular fluid reach potentially neurotoxic levels following systemic L-tryptophan loading. Brain Res..

[228] Foster A.C., Collins J.F., Schwarcz R. (1983). On the excitotoxic properties of quinolinic acid, 2,3-piperidine dicarboxylic acids and structurally related compounds. Neuropharmacology.

[229] Santamaría A., Ríos C. (1993). MK-801, an N-methyl-D-aspartate receptor antagonist, blocks quinolinic acid-induced lipid peroxidation in rat corpus striatum. Neurosci. Lett..

[230] Shear D.A., Dong J., Gundy C.D., Haik-Creguer K.L., Dunbar G.L. (1998). Comparison of intrastriatal injections of quinolinic acid and 3-nitropropionic acid for use in animal models of Huntington's disease. Prog. Neuropsychopharmacol. Biol. Psychiatry.

[231] Behan W.M., McDonald M., Darlington L.G., Stone T.W. (1999). Oxidative stress as a mechanism for quinolinic acid-induced hippocampal damage: Protection by melatonin and deprenyl. Br. J. Pharmacol..

[232] Pérez-De La Cruz V., González-Cortés C., Galván-Arzate S., Medina-Campos O.N., Pérez-Severiano F., Ali S.F., Pedraza-Chaverrí J., Santamaría A. (2005). Excitotoxic brain damage involves early peroxynitrite formation in a model of Huntington's disease in rats: Protective role of iron porphyrinate 5,10,15,20-tetrakis (4-sulfonatophenyl)porphyrinate iron (III). Neuroscience.

[233] Rodríguez-Martínez E., Camacho A., Maldonado P.D., Pedraza-Chaverrí J., Santamaría D., Galván-Arzate S., Santamaría A. (2000). Effect of quinolinic acid on endogenous antioxidants in rat corpus striatum. Brain Res..

[234] Stone T.W., Behan W.M., MacDonald M., Darlington L.G. (2000). Possible mediation of quinolinic acid-induced hippocampal damage by reactive oxygen species. Amino Acids..

[235] del Río P., Montiel T., Chagoya V., Massieu L. (2007). Exacerbation of excitotoxic neuronal death induced during mitochondrial inhibition *in vivo*: Relation to energy imbalance or ATP depletion?. Neuroscience.

[236] del Río P., Massieu L. (2008). Mild mitochondrial inhibition *in vivo* enhances glutamate-induced neuronal damage through calpain but not caspase activation: Role of ionotropic glutamate receptors. Exp. Neurol..

[237] Henneberry R.C., Novelli A., Cox J.A., Lysko P.G. (1989). Neurotoxicity at the N-methyl **-**D- aspartate receptor in energy-compromised neurons. An hypothesis for cell death in aging and disease. Ann. N.Y. Acad. Sci..

[238] Henneberry R.C., Novelli A., Vigano M.A., Reilly J.A., Cox J.A., Lysko P.G. Energy-related neurotoxicity at the NMDA receptor: A possible role in Alzheimer's disease and related disorders. Prog. Clin. Biol. Res..

[239] Greene J.G., Greenamyre J.T. (1996). Manipulation of membrane potential modulates malonate-induced striatal excitotoxicity *in vivo*. J. Neurochem..

[240] Ikonomidou C., Turski L. (1996). Neurodegenerative disorders: Clues from glutamate and energy metabolism. Crit. Rev. Neurobiol..

[241] Massieu L., García O. (1998). The role of excitotoxicity and metabolic failure in the pathogenesis of neurological disorders. Neurobiology (Bp)..

[242] Jacquard C., Trioulier Y., Cosker F., Escartin C., Bizat N., Hantraye P., Cancela J.M., Bonvento G., Brouillet E. (2006). Brain mitochondrial defects amplify intracellular [Ca^2+^] rise and neurodegeneration but not Ca^2+^ entry during NMDA receptor activation. FASEB J..

[243] Bazzett T.J., Falik R.C., Becker J.B., Albin R.L. (1996). Synergistic effects of chronic exposure to subthreshold concentrations of quinolinic acid and malonate in the rat striatum. Brain Res..

[244] Ryu J.K., Kim S.U., McLarnon J.G. (2004). Blockade of quinolinic acid-induced neurotoxicity by pyruvate is associated with inhibition of glial activation in a model of Huntington's disease. Exp. Neurol..

[245] Guidetti P., Schwarcz R. (1999). 3-Hydroxykynurenine potentiates quinolinate but not NMDA toxicity in the rat striatum. Eur. J. Neurosci..

[246] Fatokun A.A., Stone T.W., Smith R.A. (2008). Prolonged exposures of cerebellar granule neurons to *S*-nitroso-*N*-acetylpenicillamine (SNAP) induce neuronal damage independently of peroxynitrite. Brain Res..

[247] Márquez-Valadez B., Lugo-Huitrón R., Valdivia-Cerda V., Miranda-Ramírez L.R., Pérez-De La Cruz V., González-Cuahutencos O., Rivero-Cruz I., Mata R., Santamaría A., Pedraza-Chaverrí J. (2009). The natural xanthone alpha-mangostin reduces oxidative damage in rat brain tissue. Nutr. Neurosci..

[248] Sudati J.H., Fachinetto R., Pereira R.P., Boligon A.A., Athayde M.L., Soares F.A., de Vargas-Barbosa N.B., Rocha J.B. (2009). *In vitro* antioxidant activity of *Valeriana officinalis* against different neurotoxic agents. Neurochem. Res..

[249] Kumar P., Kumar A. (2009). Neuroprotective effect of cyclosporine and FK506 against 3-nitropropionic acid induced cognitive dysfunction and glutathione redox in rat: Possible role of nitric oxide. Neurosci. Res..

[250] Ubhi K., Lee P.H., Adame A., Inglis C., Mante M., Rockeinstein E., Stefanova N., Wenning G.K., Masliah E. (2009). Mitochondrial inhibitor 3-nitropropionic acid enhances oxidative modifications of alpha-synuclein in a transgenic mouse model of multiple system atrophy. J. Neurosci. Res..

[251] Pandey M., Borah A., Varghese M., Barman P.K., Mohanakumar K.P., Usha R. (2009). Strital dopamine level contributes vto hydroxyl radical generation and subsequent neurodegeneration in the striatum in 3-nitropropionic acid-induced Huntington’s disease in rats. Neurochem. Int..

[252] Medina-Navarro R., Guerrero-Linares I. (2009). Whole body hyperthermia reduced oxidative stress in the striatum of rats in an animal model of mitochondrial toxicity with 3-nitropropionic acid. Int. J. Hyperthermia.

[253] Yang L., Calingasan N.Y., Wille E.J., Cormier K., Smith K., Ferrante R.J., Beal M.F. (2009). Combination therapy with coenzyme Q10 and creatine produces additive neuroprotective effects in models of Parkinson’s and Huntington’s diseases. J. Neurochem..

[254] Kumar P., Kalonia H., Kumar A. (2009). Sesamol attenuate 3-nitropropionic acid-induced Huntington-like behavioral, biochemical, and cellular atlerations in rats. J. Asian Nat. Prod. Res..

[255] Kumar P., Kumar A. (2009). Effect of lycopene and epigallocatechin-3-gallate against 3-nitropropionic acid induced cognitive dysfunction and glutathione depletion in rat: A novel nitric oxide mechanism. Food Chem. Toxicol..

[256] Kumar P., Kumar A. (2009). Possible neuroprotective effect of *Withania somnifera* root extract against 3-nitropropionic acid-induced behavioral, biochemical, and mitochondrial dysfunction in an animal model of Huntington’s disease. J. Med. Food.

[257] Lagoa R., López-Sánchez C., Samhan-Arias A.K., Gañan C.M., García-Martínez V., Gutíerrez-Merino C. (2009). Kaempferol protects against rat striatal degeneration induced by 3-nitropropionic acid. J. Neurochem..

[258] Al Mutairy A., Al Kadasah S., Elfaki I., Arshaduddin M., Malik D., Al Moutaery K., Tariq M. (2009). Trolox ameliorates 3-nitropropionic acid -induced neurotoxicity in rats. Neurotoxicol. Teratol..

[259] Tasset I., Pérez-De La Cruz V., Elinos-Calderón D., Carrillo-Mora P., González-Herrera I.G., Luna-López A., Königsberg M., Pedraza-Chaverrí J., Maldonado P.D., Ali S.F., Túnez I., Santamaría A. (2010). Protective effect of tert-butylhydroquinone on the quinolinic acid-induced toxicity in rat striatal slices: Role of the Nrf2-antioxidant response element pathway. Neurosignals.

[260] Elinos-Calderón D., Robledo-Arratia Y., Pérez-De La Cruz V., Padraza-Chaverrí J., Ali S.F., Santamaría A. (2009). Early nerve ending rescue from oxidative damage and energy failure by L-carnitine as post-treatment in two neurotoxic models in rat: Recovery of antioxidant and reductive capacities. Exp. Brain Res..

[261] Kada S., Nakagawa T., Ito J. (2009). A mouse model for degeneration of the spiral ligament. J. Assoc. Res. Otolaryngol..

[262] Uo T., Veenstra T.D., Morrison R.S. (2009). Histone deacetylase inhibitors prevent p53-dependent and p53-independent Bax-mediated neuronal apoptosis through two disctinct mechanisms. J. Neurosci..

[263] Liot G., Bossy B., Lubitz S., Kushnareva Y., Sejbuk N., Bossy-Wetzel E. (2009). Complex II inhibition by 3-NP causes mitochondrial fragmentation and neuronal cell death via an NMDA- and ROS-dependent pathway. Cell Death Differ..

[264] Zhang X.D., Wang Y., Wang Y., Zhang X., Han R., Wu J.C., Liang Z.Q., Gu Z.L., Han F., Fukunaga K., Qin Z.H. (2009). p53 mediates mitochondria dysfunction-triggered autophagy activation and cell death in rat striatum. Autophagy.

[265] Singh S., Misiak M., Beyer C., Arnold S. (2009). Cytochrome c oxidase isoform IV-2 is involved in 3-nitropropionic acid-induced toxicity in striatal astrocytes. Glia.

[266] Kraft J.C., Osterhaus G.L., Ortiz A.N., Garriz P.A., Johnson M.A. (2009). *In vivo* dopamine release and uptake impairments in rats treated with 3-nitropropionic acid. Neuroscience.

[267] Diwakarla S., Mercer L.D., Kardashsyan L., Chu P.W., Shin Y.S., Lau C.L., Hughes M.L., Nagley P., Beart P.M. (2009). GABAergic striatal neurons exhibit caspase-independent, mitochondrially mediated programmed cell death. J. Neurochem..

[268] Wu C.L., Hwang C.S., Yang D.I. (2009). Protective effects of brain-derived neurotrophic factor against neurotoxicity of 3-nitropropionic acid in rat cortical neurons. Neurotoxicology.

[269] Wu C.L., Chen S.D., Hwang C.S., Yang D.I. (2009). Sonic hedgehog mediates BDNF-induced neuroprotection against mitochondrial inhibitor 3-nitropropionic acid. Biochim. Biophys. Res. Commun..

[270] Almeida S., Laço M., Cunha-Oliveira T., Oliveira C.R., Rego A.C. (2009). BDNF regulates BIM expression levels in 3-nitropropionic acid-treated cortical neurons. Nuerobiol. Dis..

[271] Wei Z., Chigurupati S., Bagsiyao P., Henriquez A., Chan S.L. (2009). The brain uncoupling protein UCP4 attenuates mitochondrial toxin-induced cell death: Role of extracellular signal-regulated kinases in bioenergetics adaptation and cell survival. Neurotox. Res..

